# A flavin-dependent halogenase from metagenomic analysis prefers bromination over chlorination

**DOI:** 10.1371/journal.pone.0196797

**Published:** 2018-05-10

**Authors:** Pia R. Neubauer, Christiane Widmann, Daniel Wibberg, Lea Schröder, Marcel Frese, Tilman Kottke, Jörn Kalinowski, Hartmut H. Niemann, Norbert Sewald

**Affiliations:** 1 Organic and Bioorganic Chemistry (OCIII), Bielefeld University, Bielefeld, Germany; 2 Structural Biochemistry (BCIV), Bielefeld University, Bielefeld, Germany; 3 Center for Biotechnology (CeBiTec), Bielefeld University, Bielefeld, Germany; 4 Physical Chemistry (PCIII), Bielefeld University, Bielefeld, Germany; Weizmann Institute of Science, ISRAEL

## Abstract

Flavin-dependent halogenases catalyse halogenation of aromatic compounds. In most cases, this reaction proceeds with high regioselectivity and requires only the presence of FADH_2_, oxygen, and halide salts. Since marine habitats contain high concentrations of halides, organisms populating the oceans might be valuable sources of yet undiscovered halogenases. A new Hidden-Markov-Model (HMM) based on the PFAM tryptophan halogenase model was used for the analysis of marine metagenomes. Eleven metagenomes were screened leading to the identification of 254 complete or partial putative flavin-dependent halogenase genes. One predicted halogenase gene (*brvH*) was selected, codon optimised for *E*. *coli*, and overexpressed. Substrate screening revealed that this enzyme represents an active flavin-dependent halogenase able to convert indole to 3-bromoindole. Remarkably, bromination prevails also in a large excess of chloride. The BrvH crystal structure is very similar to that of tryptophan halogenases but reveals a substrate binding site that is open to the solvent instead of being covered by a loop.

## Introduction

In many bioactive compounds, halogen substituents are an important factor for the biological activity [1]. Haloperoxidases and cofactor- and metal-free haloperoxidases (perhydrolases) were the first known halogenating enzymes. These require hydrogen peroxide for the halogenation reaction and lack substrate specifity and regioselectivity [2,3]. Flavin-dependent halogenases (FHals) play an essential role in the regioselective halogenation of natural products such as chloramphenicol [4,5], vancomycin [6,7], or cryptophycin [8,9]. Chemical halogenation of aromatic compounds often requires harsh reaction conditions using catalysts like Lewis acids and often lacks regioselectivity. FHals have the potential to overcome the drawbacks of conventional chemical approaches, as they work under more environmentally friendly conditions [10,11]. Only halide salts, oxygen, and the cofactor FADH_2_ are required for regioselective halogenation [10]. Moreover, it is possible to combine chemical synthesis with enzymatic halogenation in one-pot reactions. Frese et al. combined enzymatic halogenation of tryptophan with a subsequent Suzuki-Miyaura cross-coupling reaction leading selectively to C5-, C6- or C7-aryl-substituted tryptophan derivatives [12]. Preparative amounts of halogenated products can be obtained using immobilisation of the halogenases and their auxiliary enzymes as cross-linked enzyme aggregates (CLEAs) [13].

FHals can be divided into two major classes based on their substrate preference [14]. Halogenases belonging to variant A accept free soluble substrates. The tryptophan 7-halogenases RebH [15] and PrnA [16,17], the tryptophan 6-halogenase Thal [18] as well as the tryptophan 5-halogenase PyrH [19] belong to the most prominent members of this variant which can be subdivided into two subgroups with one group catalysing the halogenation of tryptophan and indole derivatives, whereas the other group only accepts phenol and pyrrole derivatives [20,21]. In contrast, variant B halogenases require substrates that are bound to carrier proteins. These halogenases catalyse a step in non-ribosomal peptide synthesis or polyketide synthesis. CndH and PltA are examples of enzymes that halogenate such carrier-bound tyrosyl- or pyrrolyl-residues during the biosynthesis of chondrochloren or pyoluteorin, respectively [14,22]. Most of the known FHals are of bacterial origin, but during the last years, fungal FHals have been identified as well. Rdc2, which is responsible for catalysing the halogenation of radicicol, was the first identified fungal FHal [23]. Recently, the fungal FHals, RadH and MalA, which are able to halogenate complex substrates, have been identified [24,25]. RadH is highly similar to Rdc2 on amino acid level and is able to halogenate monocillin II in radicicol biosynthesis [24]. MalA is involved in the synthesis of malbrancheamide and halogenates premalbrancheamide. It is proposed that MalA may be a part of a new class of FHals, which possess a Zn^2+^-binding C-terminus and a flexible active site for the halogenation of complex substrates [25].

Halogenases have the potential to provide a clean biocatalytic alternative to chemical halogenation. Yet, only a few FHals have been fully characterised. One of the major reasons for this is the cumbersome identification of the natural substrate that is being accepted by the enzyme, especially in case of variant B halogenases that act on carrier-bound intermediates of biosynthetic pathways. Hence, mainly FHals catalysing the regioselective halogenation of freely soluble tryptophan in different positions have been characterised until now. Based on the crystal structures of PrnA [26], RebH [27] and PyrH [21], a reaction mechanism has been suggested: FADH_2_ reacts with molecular oxygen forming a flavin-hydroperoxide (FAD-OOH). This intermediate is attacked by a halide ion, leading to the formation of hypohalous acid (HOX) within the flavin binding site. The HOX is then transferred within the enzyme through a tunnel of 10 Å in length to the substrate binding site. A conserved lysine residue (K79 in PrnA [26]) is responsible for the electrophilic aromatic substitution of the substrate [21, 26, 27]. The putative influence of an *N*-haloamine of this particular lysine residue suggested on the basis of experimental evidence on the halogenation reaction is still under debate. Besides this, a conserved glutamic acid (E346 in PrnA) [26] is essential for appropriate orientation and further activation of HOX prior to halogenation [28]. Two additional regions are highly conserved in flavin-dependent halogenases: the FAD binding module (GxGxxG) [29,30] and a WxWxIP motif suggested to prevent the enzyme from functioning as a monooxygenase [26]. These conserved regions provide a promising basis for the identification of new FHals from other organisms based on a bioinformatic approach.

Considerable effort was put into the investigation of novel halogenases in the recent years. For example, directed evolution led to more thermostable halogenases [31]. The Lewis group used structure information for switching the regioselectivity of a halogenase [32]. In 2005, Zehner and coworkers positively identified the Trp 5-halogenase, PyrH in the model organism *Strepomyces rugosporus* LL-42D005 based on conserved regions by using degenerative primers for PCR amplification [19]. Recently, Smith et al. identified a novel FHal from the metagenome of the marine sponge *Theonella swinhoei WA* that halogenates 5-hydroxytryptophan. Specific primers were used for identification of this novel halogenase [33]. Bioinformatic analyses for the identification of novel enzymes have so far been successfully carried out for different biotechnologically relevant enzymes, albeit not for FHals. For example, an unknown laccase-like enzyme [34] and an active chitinase [35] have been identified from metagenomic data sets by applying bioinformatics analyses. For the identification an adapted Hidden-Markov-model (HMM) was applied, which detected conserved regions of the particular enzymes [34,35].

Halides like chloride and bromide are present in high concentrations in marine habitats. Although the chloride concentration (19.345 ‰) is much higher than the bromide concentration (0.066 ‰) [36], many brominated natural products have been found in marine organisms [37]. It was reported that terrestrial halogenases mainly use chloride while marine halogenases tend to prefer bromide. Therefore, the ocean is a beneficial habitat for the evolution of brominating enzymes [20]. However, many of these potential enzymes are encoded by genes originating from microorganisms that are uncultivable under standard laboratory conditions. In this study, different marine metagenomes were screened for FHals by employing a HMM that is based on the PFAM tryptophan halogenase model (Trp_halogenase, PF04820). We expected to find novel enzymes from cultivable as well as from uncultivable organisms using metagenomic data sets in order to increase the scope of possible halogenases.

## Materials and methods

### Materials

The chemicals and solvents were obtained, unless otherwise noted, from commercial suppliers in highest purity suitable for analytical applications (*p*. *a*.).

The plasmid vector pET-21_ADH encoding for alcohol dehydrogenase was kindly donated by Prof. Dr. Werner Hummel and the plasmid pClBhis-PrnF encoding for the flavin reductase in *Pseudomonas fluorescens* was a gift from Prof. Dr. Karl-Heinz van Pée. The plasmid pGro7 for the chaperone GroEL-GroES was purchased from TaKaRa Bio Inc. The plasmid pETM-11 was obtained from Gunter Stier (EMBL). Competent cells *E*. *coli* DH5α and *E*. *coli* BL21 (DE3) were obtained from Novagen. ThermoFisher GeneArt synthesised the flavin-dependent halogenase genes *brvH*.

### Analytical reversed-phase high performance liquid chromatography (RP-HPLC)

For analytical reversed-phase high performance liquid chromatography, three different methods were used. By using method A, reactions were monitored using a Thermo Scientific Accela 600 with Hypersil GOLD 3 μm from Thermo Scientific (150 × 2.1 mm, eluent A: H_2_O/CH_3_CN/TFA = 95:5:0.1, eluent B: H_2_O/CH_3_CN/TFA = 5:95:0.1, flow rate 700 μl/min with a linear gradient from 0–100% of eluent B over 5 minutes, for 1 minute with eluent B, then in half a minute going back to 100% eluent A and staying with 100% A for 2 minutes).

In method B, reactions were monitored using a Thermo Scientific Accela 600 with NUCLEOSHELL^®^ RP 18 column 18.5 μm from Macherey-Nagel (150 × 2.1 mm, eluent A: H_2_O/CH_3_CN/TFA = 95:5:0.1, eluent B: H_2_O/CH_3_CN/TFA = 5:95:0.1, flow rate 900 μl/min, first minute 100% eluent A, then a linear gradient from 0–100% eluent B in 5 minutes, 1 minute at 100% B and in a half minute going back to 100% A. At the end staying for 2 minutes with eluent A).

Based on method C, reactions were monitored using a Shimadzu Nexera XR Luna^®^ 3 μm C18(2) 100 Å, LC Column from Phenomenex (100 × 2 mm, eluent A: H_2_O/CH_3_CN/TFA = 95:5:0.1, eluent B: H_2_O/CH_3_CN/TFA = 5:95:0.1, flow rate 500 μl/min isocratic with 35% of eluent B over 5 minutes).

For the reactions monitored by method D a column from Phenomenex (XR Luna^®^ 3 μm C18(2) 100 Å, LC column, 100 × 2 mm) was used. Eluent A: H_2_O/CH_3_CN/TFA = 95:5:0.1, eluent B: H_2_O/CH_3_CN/TFA = 5:95:0.1, flow rate 650 μl/min isocratic with 35% of eluent B over 7 minutes.

### Preparative reversed-phase high performance liquid chromatography (RP-HPLC)

For purification of products a preparative HPLC (Merck-Hitachi LaChrom) with Thermo Fisher Hypersil Gold 8 μm column (250 × 21.2 mm, eluent A:H_2_O/CH_3_CN/TFA = 95:5:0.1, eluent B: H_2_O/CH_3_CN/TFA = 5:95:0.1, flow rate 10 mL/min with a linear gradient from 0–100% B over 45 minutes) was used.

### Gas chromatography—Mass spectrometry (GC-MS)

For GC-MS analysis the gas chromatograph Trace GC Ultra (ThermoScientific) with a VF-5 column 0.25 μm (30 m × 0.25 mm, 5% diphenylsiloxan, 95% dimethylsiloxan was employed; for mobile phase helium was used with temperature gradient of 5 °C/min from 80 °C to 325 °C) and the mass spectrometer ITQ900 from ThermoFinnigan (20 measurements per minute, 50–750 m/z) were used.

### Nuclear magnetic resonance (NMR) spectroscopy

NMR spectra were recorded on a Bruker DRX-500 spectrometer (^1^H: 500 MHz, ^13^C: 126 MHz). Chemical shifts are reported relative to residual solvent peaks (DMSO-*d*_*6*_: ^1^H: 2.5 ppm; ^13^C: 39.5 ppm)

## General methods

### Metagenomic analysis for the detection of flavin-dependent halogenases

All metagenomic data were obtained from the iMicrobe.us or gold.jgi.doe.gov databases. A two-step approach as recently described [34] with some modifications was used for the construction of a HMM for the detection of flavin-dependent halogenases. In the first step, the PFAM model for tryptophan halogenase (PF04820) was used as basis. To improve the initial model, conserved sequences were collected by applying BLAST [38] using known protein sequences of already characterized FHals with a threshold of 50% sequence identity und 90% query coverage as references. The obtained sequences were aligned using MUSCLE [39] and the alignment was processed manually. Finally, a halogenase HMM was generated based on the initial HMM for tryptophan halogenase and the obtained alignment using the HMMER3 package [40]. Assembly of metagenomic data was performed by applying MEGAHIT [41]. For gene prediction, the tool Prodigal [42] was used and the predicted genes were filtered based on their completeness and their match to the model. Completeness means that the gene contains a start and stop codon, conserved regions are present and the size of the genes fits the size of known halogenases. For example, the *rebH* gene contains 1593 base pairs, corresponding to 530 amino acids in length [15]. The predicted and translated genes were compared against the halogenase HMM using the HMMER3 software package. Only genes with an e-value < 1x10^-150^ were used for further analyses.

### Phylogenetic analyses of the positive halogenases hits

For phylogenetic analyses MEGA7 [43] was used. First, a protein alignment (MUSCLE) was performed and then the phylogenetic tree was constructed based on neighbour joining (NJ) method and a bootstrap of 1000.

### Vector preparation, heterologous protein expression

BrvH has been identified in course of the bioinformatic approach explained above. The gene *brvH* was identified in Botany Bay metagenome (2016 iMicrobe, CAM_SMPL_001699). The gene of *brvH* was codon optimised for *E*. *coli* and extended with restriction sites (*Nde*I and *BamH*I) for further cloning. The synthetic gene was obtained from Invitrogen GeneArt. The gene was cloned into pET-28a vector and transformed to *E*. *coli* BL21 (DE3) pGro7. The pGro7 vector codes for the expression of a chaperone system GroEL-GroES. An overnight preculture (37 °C, 150 rpm) was used to inoculate a culture in LB medium containing the appropriate antibiotics (60 μg/mL kanamycin; 50 μg/mL chloramphenicol). This culture was grown (37 °C, 150 rpm) up to an OD_600_ of 0.5. Protein expression was then induced with isopropyl-β-D-thiogalactopyranoside (IPTG) (0.1 mM) and l-arabinose (2 g/L) and the cells were cultivated at 25 °C, 150 rpm for 22 h. Afterwards, cells were harvested by centrifugation (3220 × *g*, 30 min), washed with 100 mM Na_2_HPO_4_ buffer (pH7.4) and stored at -20 °C.

For protein crystallisation, the *brvH* gene was cloned into a pETM-11 vector. This vector includes a hexahistidine tag, followed by a tobacco etch virus (TEV) protease cleavage site. The plasmid was transformed into *E*. *coli* BL21 (DE3). An overnight preculture (30 °C, 100 rpm) was used to inoculate a culture in LB medium containing 30 μg/mL kanamycin. The culture was grown (37 °C, 100 rpm) to an OD_600_ of 0.7 and protein expression was induced using 0.1 mM IPTG. After 18 h of expression at 20 °C, the cells were harvested by centrifugation (see above), and the cell pellet washed with PBS and frozen at -20 °C.

### Site-directed mutagenesis of *brvH*

A PCR was carried out to mutate the conserved lysine residue to an alanine (K83A). The following primers were designed:

BrvH_K83A: GCAACCCAGGCAACCTGTGCGCTGGGTATTCGTTTT
BrvH_K83A-rev: AAAACGAATACCCAGCGCACAGGTTGCCTGGGTTGC

For the PCR, 70 ng of vector DNA (brvH_pET-28a), 125 ng of each primer, 2.5 U/mL Pfu polymerase (Promega, Germany), 1 × reaction buffer and dNTP mix (200 μM of each nucleotide) were used. The PCR program is shown in Table 1.

**Table 1:** Program for the site-directed mutagenesis PCR.

Cycles	Temperature [°C]	Minutes
1	95	0.5
16	95	0.5
	55	1
	68	7
1	68	5

Afterwards parental DNA was digested by incubating the PCR product with DpnI restriction enzyme (10 U/μl) at 37 °C. After 1 hour, the mutated DNA was transformed into *E*. *coli* DH5α (DE3). Protein expression and purification was identical to the non-mutated BrvH.

### Protein purification

For protein isolation, the cells from 1.5 L cultivation were thawed, suspended in 30 mL 100 mM Na_2_HPO_4_ buffer (pH7.4) and lysed with the French Press (3 times 1000 psig). Soluble protein was separated from insoluble compounds by centrifugation (10000 × *g*, 30 min, 4 °C).

A HisTALON matrix was employed for purification. The elution of the bound enzymes was carried out with imidazole (300 mM imidazole, 100 mM Na_2_HPO_4_, 300 mM NaBr, pH 7.4) and resulting enzymes were collected in 0.5 mL fractions. Afterwards fractions with pure enzyme were pooled and a desalting column (HiTrap^®^ Desalting (GE Healthcare)) was used to remove the imidazole and to change the buffer (50 mM Na_2_HPO_4_, 50 mM NaBr, pH 7.4).

For protein crystallisation, purification included Ni^2+^-affinity chromatography, ion exchange (IEX) and size exclusion chromatography (SEC). The cells were first resuspended in a lysis buffer (25 mL PBS containing cOmplete^™^ protease inhibitor (EDTA-free; Merck, Germany) and 20 μg/mL DNase I) and lysed using a French Press (120 MPa). Soluble protein was obtained via centrifugation (16000 x *g*, 30 min, 4 °C) and captured via Ni^2+^-NTA affinity chromatography. Bound protein was eluted with imidazole (20 mM Tris 20 mM NaCl 200 mM imidazole pH 8) and an IEX of the protein-containing fractions was performed using an ÄKTA Purifier (self-packed Source 15 Q column (column volume (CV) = 7 mL; GE Healthcare, UK); 1 mL/min; gradient from 0 to 500 mM NaCl in 20 mM Tris pH 8 over 20 CV; fractions of 2 mL were collected). The FHal eluted in a single peak and was then subjected to an overnight TEV protease digest (1 mg TEV per 50 mg of BrvH) at 20 °C in order to remove the His_6_-tag. In a following Ni^2+^-NTA chromatography, the cleaved protein was obtained in the flowthrough and washing fractions and concentrated using a VivaSpin column (10000 MWCO; Sartorius, Germany). For SEC, a 16/60 Superdex 200 column (flow rate 1 mL/min; GE Healthcare, Germany) connected to an ÄKTA purifier at 4 °C was used (buffer 10 mM Tris 20 mM NaCl, pH8; fractions of 2 mL were collected). Eluted protein was concentrated to 22.3 mg/mL and stored at -80 °C prior to crystallisation.

### Cofactor regeneration system

The cofactor regeneration system consisting of flavin reductase (PrnF) and alcohol dehydrogenase (ADH) was generated as published previously [10].

### Crystallisation and structure determination of apo BrvH

BrvH was diluted to 10 mg/mL for crystallisation. Crystallisation conditions were screened using the JCSG Core IV Suite (Qiagen, Germany). Stacks of very thin plates were obtained after two weeks in 1 M Na/K tartrate, 0.1 M MES pH 6.0 with a drop size of 2 μL and a ratio of protein:reservoir solution of 1:1. Optimisation did not yield single crystals. However, using streak seeding, single plates could be obtained in 0.95 M Na/K tartrate 0.1 M MES pH 6.0 after one day. Prior to data collection, six-day-old crystals were transferred into the crystallisation solution supplemented with 20% glycerol and vitrified in liquid nitrogen. Data were collected at the ESRF, Grenoble, using the ID30 A-3 beamline (1800 frames; oscillation angle 0.15°; x-ray wavelength 0.9677 Å).

Raw data were processed with XDS and scaled with XSCALE [44]. The data were imported into CCP4 and merged with aimless [45] for structure solution via molecular replacement using Phaser (ensemble: PrnA, seq. identity 34%; PyrH, seq. identity 36%; RebH, seq. identity 33%; SttH, seq. identity 36%; [46]). An initial model was built by PHENIX AutoBuild [47] and further improved via manual building using COOT [48]. For crystallographic refinement of interim models, Refmac5 was used [49]. Validation with MOLPROBITY [50] was part of the iterative building process. The statistics of the final model are presented in S1 Table and the crystal structure was deposited in the protein data bank (PDB ID 6FRL). Figures were generated in PyMOL [51].

### FAD reconstitution

For FAD reconstitution, the purified enzyme was incubated in FAD buffer (50 mM Na_2_HPO_4_, 150 mM NaBr, 1 mM FAD, pH 7.4) over night at 4 °C, 5 rpm. A desalting column (HiTrap^®^ Desalting (GE Healthcare)) was used to change the buffer. After incubation overnight, BrvH was washed with buffer (50 mM Na_2_HPO_4_, 50 mM NaBr, pH 7.4) to remove free FAD using a filter device (Amicon, Ultra 4) with a 50 kDa cutoff. UV/vis spectra of the sample were measured in a quartz cuvette (Suprasil, Hellma) with a path length of 1 cm using a Shimadzu UV-2450 spectrometer.

### Assay conditions

The activity assays of PrnF and ADH were carried out as described by Frese et al. [10].

The activity assays with the novel flavin-dependent halogenases were carried out with cell lysate containing the enzymes (250 μl in 1 mL) with 1 μM FAD, 100 μM NAD, 100 mM NaBr/NaCl, PrnF (2.5 U/mL) and ADH (2 U/mL). Indole was dissolved in isopropanol to give a final substrate concentration of 1 mM and 5% solvent, respectively. When tryptophan was used as substrate, it was dissolved in water and isopropanol was added to a final concentration of 5% (v/v). Assays were incubated for 48 h at 25 °C, 300 rpm. For the activity assays using purified enzyme, a final enzyme concentration of 1.125 mg/mL was present in the reaction mixture, while the other conditions given above were unmodified. The activity assays were carried out in 1 mL or 0.5 mL volume, which was filled up with 100 mM Na_2_HPO_4_ (pH 7.4). If appropriate, catalase (650 U/mL) was added to 500 μl reaction mixture of the activity assays to remove peroxide intermediates.

### Indole conversion on large scale by immobilised enzymes

Cross-linked enzyme aggregates (CLEAs) as described by Frese et al. [13] were used for conversion of higher amounts of indole for NMR analyses. First, the *E*. *coli* BL21_pGro7 cells containing BrvH from a 1.5 L cultivation batch were disrupted using French Press and centrifuged as described above. 2.5 U/mL PrnF (flavin reductase) and 1 U/mL alcohol dehydrogenase (ADH) were added to the supernatant. This protein mixture was then separated into two equal parts because one cell pellet was used for two CLEA reactions. The proteins were precipitated by adding saturated ammonium sulfate solution in a tube rotator for 1 h at 4 °C. Glutaraldehyde was added to a final concentration of 0.5% and the mixture was further rotated for 2 h. Finally, the produced CLEAs were centrifuged and three times washed with 100 mM Na_2_HPO_4_ buffer (pH7.4). For the biocatalysis with CLEAs 1.5 mM substrate, 1 μM FAD, 100 μM NAD, 15 mM Na_2_HPO_4_, 30 mM NaBr and 5% (v/v) isopropanol were employed. The pH was adjusted with phosphoric acid to pH 7.4. CLEAs were added to the reaction solution in a final volume of 500 mL and incubated for up to 10 days (depending on the conversion) at 25 °C, 150 rpm.

## Results and discussion

### Selection of metagenomic data sets, construction of the HMM, bioinformatic analyses and phylogenetic reconstruction of the identified FHal genes from Botany Bay to known FHal genes

**Fig 1:**
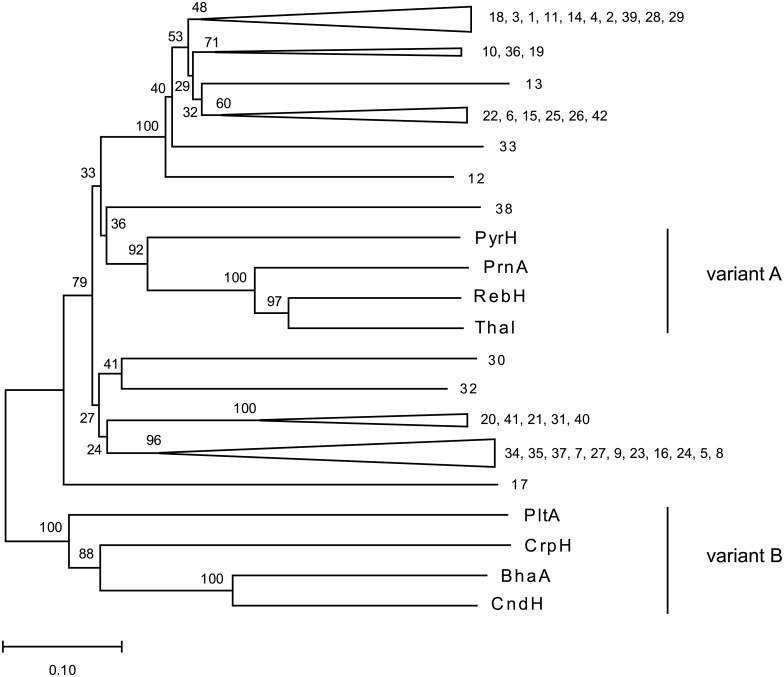
Phylogenetic tree of positive detected FHals from the Botany Bay metagenome in comparison to known FHals on amino acid level. The tree was constructed using neighbour joining method, bootstrap 1000, with the alignment based on amino acids by MEGA7 [43] and MUSCLE [39]. 1–42: positive FHal hits from the Botany Bay metagenome; PyrH, PrnA, RebH, Thal: Tryptophan halogenases belonging to variant A and accept free substrate; PltA, CrpH, BhaA, CndH: FHals belonging to variant B and require carrier bound substrate. The amino acid sequences are shown in S3 Table.

For the identification of novel FHals, we analysed eleven metagenomes (S2 Table). For screening, we established a HMM based on a two-step approach for tryptophan halogenases like recently described by Ausec et al. [34] with some modifications. After that, the halogenase HMM was applied to identify FHals within the metagenomic data sets. By applying the improved HMM model, several potential FHal genes were identified within the screened metagenomes. In total, 254 predicted FHal genes were identified, but only 77 were shown to be complete, meaning that the gene contains a start and stop codon, conserved regions are present and its size fits to the size of known halogenases. For example, the *rebH* gene contains 1593 base pairs, corresponding to 530 amino acids in length. The highest number of complete genes (42 in total) was identified from the Botany Bay metagenome (CAM_SMPL_001699). The two variants can be distinguished by multiple sequence alignment, which can be visualised by a phylogenetic tree. We built a phylogenetic tree with the predicted FHals from the Botany Bay metagenome in comparison to known FHals belonging to variant A and B on amino acid level. The tree was constructed via MUSCLE alignment and neighbour joining with a bootstrap of 1000 replicates using MEGA7 [43] (Fig 1). In this phylogenetic tree, the FHals of both classes cluster into two distinct clades consisting of variant A and variant B FHals. Remarkably, all predicted genes cluster in the clade of tryptophan halogenases, but only gene 38 clusters within the cluster of tryptophan halogenases. Some of the predicted FHal genes cluster more distantly from the tryptophan halogenases (5, 7, 8, 9, 16, 20, 21, 23, 24, 27, 30, 31, 32, 34, 35, 37, 40, 41). The predicted gene 17 seems to be an outgroup, because it is more distant from both the variant A and the variant B FHals. The fact that all predicted genes cluster closer to the tryptophan halogenases may be caused by the selection criteria since our HMM consists of the PFAM model of tryptophan halogenases. Moreover, the e-value and the gene size resembling the length of known tryptophan halogenases were used as criteria for selection. Weichold et al. postulated that variant B FHals seem to be around 70 amino acids shorter in comparison to variant A FHals [52]. Therefore, it can be assumed that our selection criteria rejected predicted genes belonging to variant B FHals and for this reason from 254 predicted genes only 77 were assigned as complete.

### Selection, expression and activity assay of a predicted FHal gene from Botany Bay

We decided to investigate one predicted FHal gene from the Botany Bay metagenome, which clusters close to tryptophan halogenases within the phylogenetic tree. We chose predicted gene 12 (1530 bp), which shows high similarities to tryptophan halogenases on amino acid level, but interestingly forms its own clade within the phylogenetic tree.

Sequence comparison of gene 12 using Blastp and the nr database resulted in one hit with 100% identity on amino acid level to an annotated putative tryptophan halogenase from *Brevundimonas* BAL3 (NCBI: Acc. # EDX81295.1), but no enzymatic activity of its gene product had been described. *Brevundimonas* BAL3 is a gram-negative alphaproteobacterium that was isolated from marine habitat (GOLD Project ID: Gp0006177). To the best of our knowledge, no halogenated product is known from this organism to date. AntiSMASH analysis of the genome of *Brevundimonas* BAL3 revealed one gene cluster leading to bacteriocin [53]. Since it is most likely that the predicted FHal gene is from *Brevundimonas BAL3*, we designated gene 12 as *brvH*.

**Fig 2:**
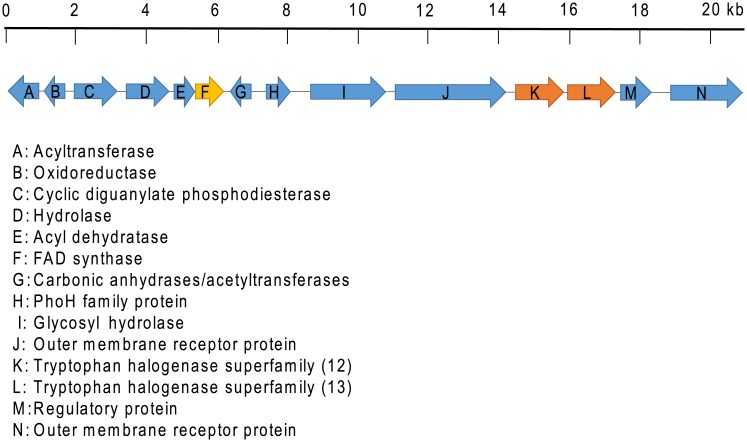
Gene cluster in the vicinity of gene 12 (K) in the Botany Bay metagenome. Upstream to the gene 12 (K), another FHal, 13 (L) was identified.

Gene cluster analysis revealed another FHal gene 13 (1542 bp), located upstream of gene 12 (Fig 2). Gene 12 and 13 share 42.7% pairwise identity to each other.

A multiple sequence alignment based on amino acid sequences was carried out to compare BrvH with the tryptophan halogenases RebH, Thal and PyrH. The multiple alignment showed that BrvH contains all three conserved amino acid regions that were used for identification: (i) the FAD binding module, GxGxxG (amino acids (aa) 16–21), (ii) the conserved lysine residue (K83) and (iii) the conserved domain WxWxIP (aa 275–280). We decided to proceed with *in vitro* assays of BrvH to show halogenase activity and identify possible substrates. The synthetic gene *brvH* (codon optimised for *E*. *coli*) was cloned into the expression vector pET-28a. The gene was heterologously expressed in *E*. *coli* with coexpression of the chaperones GroEL-GroES. This resulted in adequate production of BrvH, which was obtained in soluble form and purified using an N-terminal His_6_-tag with immobilised metal ion chromatography (IMAC; S1 and S2 Figs).

**Fig 3:**
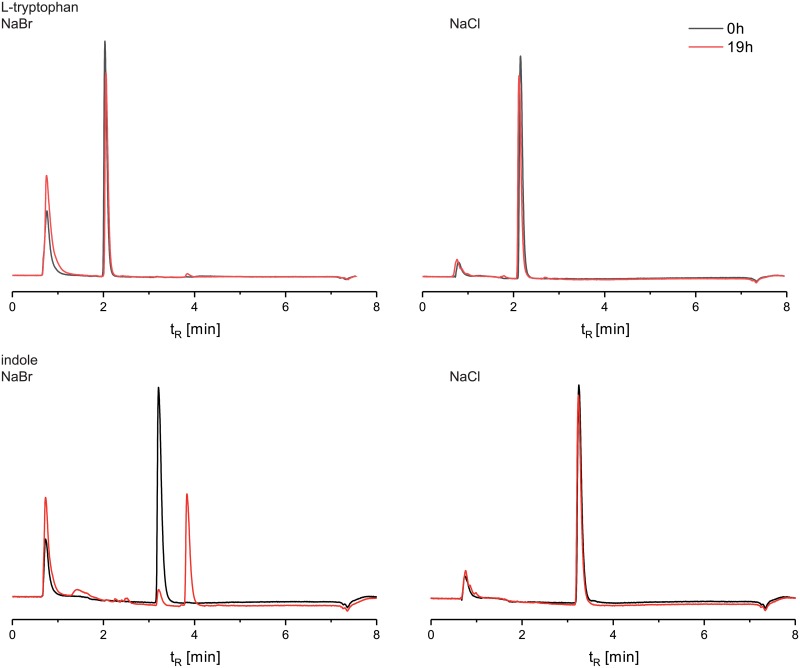
HPLC traces of the enzymatic halogenation of l-tryptophan (1 mM) and indole (1 mM) by BrvH (in cell lysate for 19 h in presence of 100 mM NaCl or NaBr). l-tryptophan (t_R_: 2.2 minutes) is not being halogenated, while Indole (t_R_: 3.2 minutes) is fully converted within 19 h to bromoindole (t_R_: 3.8 minutes) (HPLC method A).

Because our HMM is based on tryptophan halogenases and BrvH shows high similarities to these enzyme class, we suspected that tryptophan is halogenated by BrvH. However, substrate tests with cell lysate containing BrvH led to no halogenation of tryptophan, neither in presence of chloride nor in presence of bromide. Therefore, other substrates with an indole moiety, for example 5-hydroxytryptohan, tryptophol, indole-3-propionic acid, indole-3-acetonitrile, and indole were tested. However, only indole was found to be accepted as substrate (Fig 3).

The bromoindole product was further investigated by GC-MS measurements (S3 Fig) as well as NMR analyses. COSY and HMQC experiments unambiguously proved that bromination took place in position C3 of the indole ring (S4–S7 Figs). The position of halogenation may be the reason for the non-acceptance of tryptophan. Indole and tryptophan possess an identical basic structure, but the C3 position of the indole ring is occupied in tryptophan.

**Fig 4:**
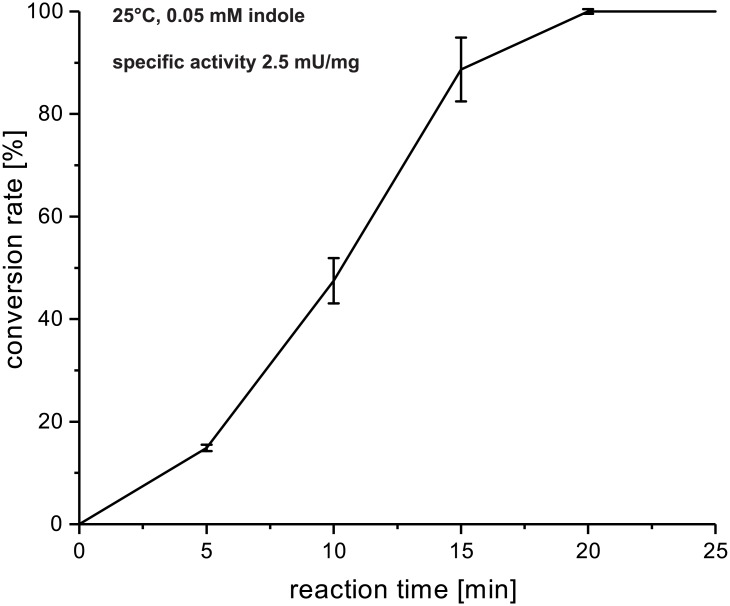
Time course of the conversion of 0.05 mM indole by BrvH with NaBr over 25 minutes at 25 °C. The conversion rate was identified via RP-HPLC by determining the ratio of the peak areas of indole and bromoindole. The specific activity was defined between 0 and 15 minutes reaction time.

The activity of BrvH underlines the feasibility of our method for the identification of novel FHals, showing that our HMM is able to identify active FHals only based on conserved regions. Low specific activity of 2.5 mU/mg and a turnover number of 39.6 lead to the conclusion that indole may not be the natural substrate of BrvH (Fig 4).

Nevertheless, it is challenging to find a suitable substrate for the identified halogenase if no halogenated natural product of the related organism is known.

**Fig 5:**
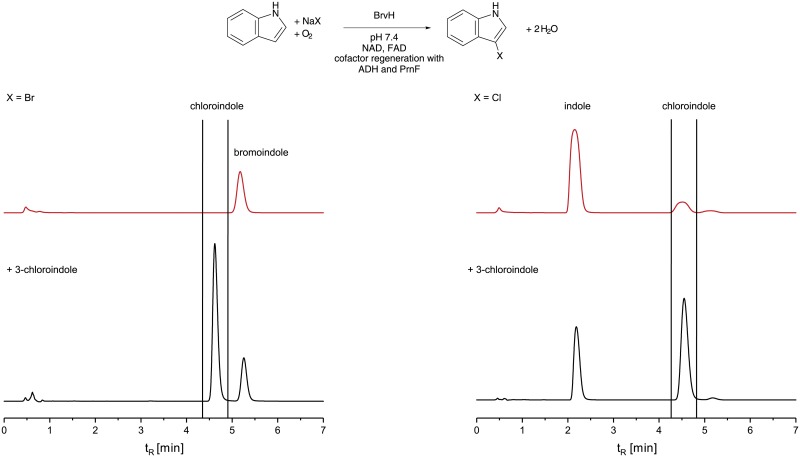
HPLC traces of enzyme products of BrvH incubated with indole and NaBr/NaCl and the same products spiked with commercially available 3-chloroindole. BrvH incubated with indole and NaBr leads to bromoindole (t_R_: 5.2 min) and incubated with NaCl leads to chloroindole (t_R_: 4.6 min). Both enzyme products spiked with 3-chloroindole (t_R_: 4.6 min) show that the halogenated product of BrvH incubated with indole and NaCl has the same retention time with commercially 3-chloroindole (HPLC method D).

Noteworthily, BrvH prefers bromide to chloride as halide source. In comparison, pure BrvH fully converts indole to bromoindole within 48 h while chlorination under identical conditions proceeds to 8.4% (S8 and S9 Figs). We need to state that we could not observe the mass of chloroindole in GC-MS measurements due to insufficient concentrations of produced chloroindole. Therefore, we obtained 3-chloroindole commercially and spiked it into the samples of the product obtained upon incubation of indole and NaCl with BrvH (Fig 5) giving the same retention times, while the halogenation product of indole with NaBr has a different retention time. This provides evidence that the halogenated product incubated with NaCl is chloroindole.

**Fig 6:**
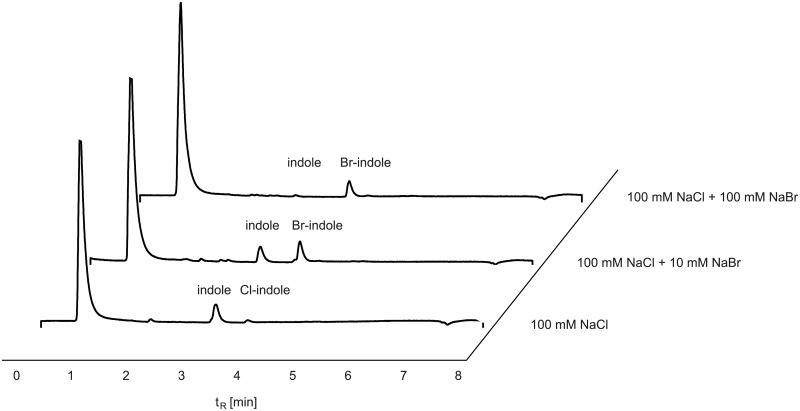
HPLC traces of the halogenation of indole with BalH in excess of NaCl (100 mM) and three different concentrations of NaBr (0 mM, 10 mM, 100 mM). Even in 10 times higher concentrations of chloride over bromide, bromide is the preferred halide source (HPLC method A).

Most FHals catalyse chlorination as well as bromination, but chlorination activity usually is higher compared to bromination activity [52]. Bmp2 and Bmp5 are the only known FHals, which only catalyse bromination but not chlorination [54]. Interestingly, BrvH preferentially brominates its substrate but is also able to chlorinate in low amounts. BrvH prefers the bromination to chlorination even in excess of chloride as halide source. The HPLC chromatogram of the sample with tenfold excess of NaCl to NaBr (Fig 6) shows that small amounts of chloroindole (t_R_: 3.75 min) as well as bromoindole (t_R_: 3.8 min) were formed. Hence, chlorination is much less efficient than bromination and even in ten times excess of NaCl, NaBr is preferred.

In order to confirm that BrvH is a flavin-dependent halogenase we performed different experiments for evaluation. Besides FHals, haloperoxidases and perhydrolases are able to directly introduce halogen substituents into organic compounds in the presence of hydrogen peroxide [2,3], albeit with diminished regioselectivity.

Hydrogen peroxide was enzymatically removed *in situ* from the reaction mixture by adding catalase. Catalase enzymatically decomposes hydrogen peroxide into water and oxygen [55]. BrvH halogenates indole even in the presence of active catalase (S10 Fig). Hence, the halogenation is not dependent on free hydrogen peroxide. Furthermore, a bromoperoxidase assay [56] gave evidence that BrvH is not a bromoperoxidase. Replacement of the lysine residue in position 83 that is conserved in FHals by alanine led to complete loss of enzyme activity (S11 Fig). Furthermore, we were able to show that FAD binds to the enzyme using UV/vis spectroscopy (S12 Fig). Upon binding of FAD to BrvH the band at 450 nm shows a slight shift to 448 nm while the band around 373 nm is shifted to around 360 nm. This latter shift is caused by the changed environment of FAD inside the protein compared to the polar and strongly hydrogen-bonded environment in solution [57,58]. All these experiments support BrvH being a FHal.

### Structure determination of BrvH

The observation that BrvH preferentially brominates in combination with the sequence similarity to tryptophan halogenases but the lack of halogenated tryptophan raised the question whether these characteristics could be explained by examining the 3D structure of the enzyme.

**Fig 7:**
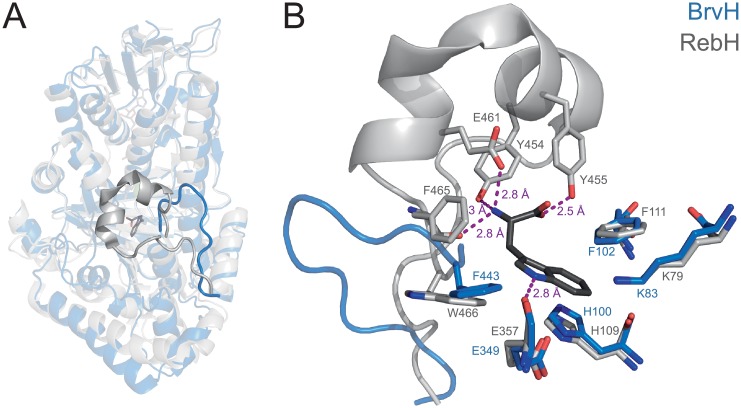
Structure of apo BrvH compared to RebH bound to FAD, Trp and Cl^-^. **A:** The structural alignment was made in PyMOL based on the conserved “box” domain of the two halogenases (BrvH: 6–99, 162–416, 494–502 vs. RebH: 2–98, 167–426, 519–528; PDB ID 2oa1). FAD, Trp (both dark grey) and Cl^-^ (green) are present only in the structure of RebH. BrvH (blue) and RebH (grey) are structurally very similar. A major difference is found in the substrate binding site, which is accessible to the solvent in BrvH, while it is covered by a longer loop in RebH. **B:** Empty substrate binding site of BrvH overlaid with the tryptophan binding site of RebH containing Trp (dark grey). Several of the RebH residues directly contacting the bound Trp are shown as grey sticks along with the corresponding residues of BrvH shown in blue. The loops that are shown correspond to those in A. While the residues contacting the indole ring are conserved, those residues that form hydrogen bonds to the carboxylate and the α-amino group of Trp in RebH are not present in BrvH. K83 and E349 of BrvH correspond to the amino acids shown to be important for the catalytic mechanism in other halogenases (K79 and E357 in RebH).

The structure of apo BrvH without any bound ligands was determined to a resolution of 2.5 Å (Fig 7A). There are two chains in the asymmetric unit with no major differences between the chains. BrvH eluted as a dimer from gel filtration. In the crystal, it forms a dimer very similar to that of tryptophan halogenases like PrnA or RebH. BrvH was modelled nearly completely, except for a few terminal residues and residues 46–51, a loop involved in FAD binding that can adopt various conformations in other FHals and is disordered in apo BrvH.

The substrate binding site is only partly conserved between BrvH and RebH. The region that is close to the active site lysine K83 (K79 in RebH) is highly similar in both proteins. Among the residues contacting the six-membered ring of the indole, there is only a single substitution (BrvH: S447 vs. RebH: N470). More variation is present among residues contacting the five-membered indole ring and the Cβ atom (BrvH: M442, F443 vs. RebH: F465, W466). The biggest difference maps to the loop that covers the tryptophan binding site in RebH and contains the amino acids (Y454, Y455, E461, F465) that form hydrogen bonds with the amino and carboxy group of the substrate (Fig 7B). There are no equivalent residues in BrvH, as a short loop (residues 432–444) replaces its much longer counterpart in RebH (residues 440–467). As a consequence, the substrate binding site of BrvH is noticeably more open than that of tryptophan halogenases and the ε-amino group of the active site lysine K83 is directly accessible to the solvent (S13 Fig).

The lack of side chains to keep the amino and carboxy group of tryptophan in place could potentially explain why BrvH does not accept tryptophan as substrate. Instead, BrvH may be able to convert larger substrates. The openness of the substrate binding site and the absence of large non-polar patches–which would point to a variant B halogenase [14]–resemble the structure of MibH [59], a tryptophan halogenase that chlorinates tryptophan only when the latter is part of the enzyme’s cognate substrate peptide (deschloro NAI-107).

**Fig 8:**
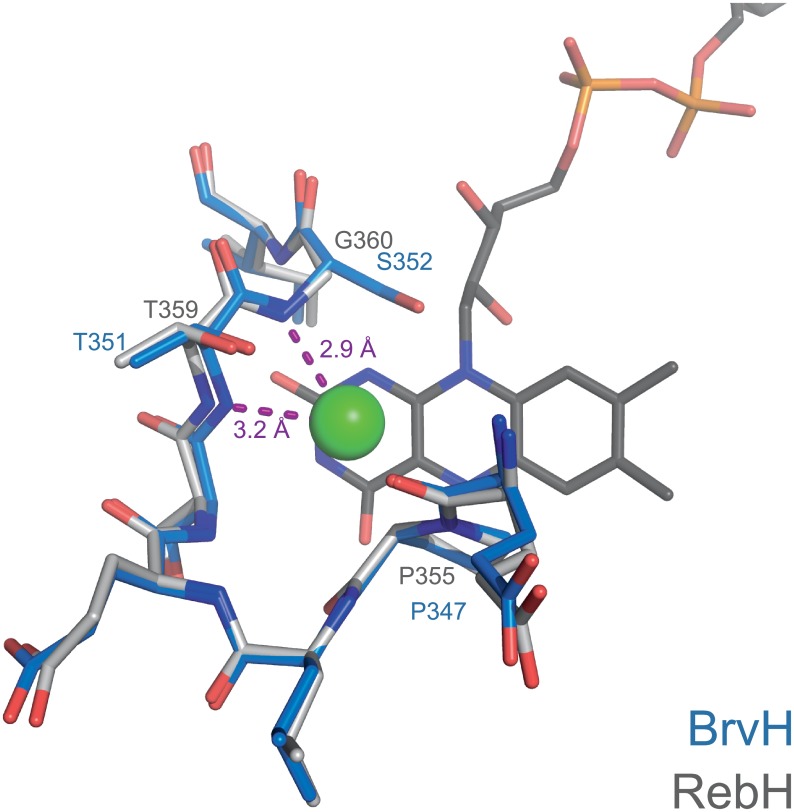
Comparison of the halide binding site in RebH and BrvH. FAD (dark grey) and Cl^-^ (green) are present only in the structure of RebH. Coordination of the halide takes place via the amide nitrogens of the backbone. Whilst the halide binding motif in RebH (grey) is T359, G360 in BrvH (blue) we see a T351, S352 at the same site. However, the amino acid exchange does not seem to alter the overall structure of the halide binding site, thus not giving a definite hint as to why BrvH would preferentially brominate.

As for the halide specificity, no definite conclusions can be drawn. The structure does not contain FAD and there is no halide bound at the assumed halide binding site, the amide nitrogens of T351 and S352 (T359 and G360 in RebH). The loop containing the halide binding motif of BrvH structurally resembles that of other FHals and its sequence is conserved between BrvH and RebH except for a single substitution (BrvH: S352 vs. RebH: G360; Fig 8). Thus, the halide specificity cannot readily be explained by the structure of the empty halide binding site.

## Conclusion

It is possible to identify FHals on genome level using bioinformatic analyses by employing a Hidden-Markov-model (HMM) based on the PFAM model for tryptophan halogenases. Several novel putative FHal genes were successfully identified from eleven metagenomes. From the 77 identified complete genes 42 originated from a metagenome from Botany Bay/Australia. Phylogenetic analysis based on FHals belonging to variant A and B halogenases in comparison to the identified genes from the Botany Bay metagenome revealed that the phylogenetic tree clusters in two distinct clades. One consists of the variant A halogenases including tryptophan halogenases and one of the variant B FHals. Interestingly, all predicted genes cluster within the tryptophan halogenases and seem to be phylogenetically closer to them than to variant B FHals. It is assumed that the predicted halogenase genes belong to variant A FHals and catalyse the halogenation of free substrates. Only the putative halogenase gene 38 clusters within the clade of tryptophan halogenases and might be a tryptophan halogenase. The other predicted genes are similar to tryptophan halogenase genes, but the corresponding enzymes may accept different substrates like BrvH (gene 12). The putative halogenase gene *brvH* from *Brevundimonas* BAL3 was further investigated *in vitro*. We were able to show that the gene product forms an active enzyme and catalyses the halogenation of free indole in C3 position. Tryptophan, however, was not halogenated. This behaviour might be explained by the lack of residues that could form hydrogen bonds to the amino and carboxy group of tryptophan in the substrate binding site of BrvH. The low specific activity towards indole suggests that indole may not be the natural substrate of BrvH. In addition, the structure of the active site could allow larger substrates to enter and possibly be halogenated. Interestingly, BrvH possesses an intriguing selectivity for bromide over chloride in halogenation that, however, cannot be directly explained from the crystal structure. Bromide is the preferred halogen source even in the presence of tenfold excess of chloride to bromide. This distinguishes BrvH from other known FHals, as these enzymes show higher activities in chlorination compared to bromination with Bmp2 and Bmp5 being the only FHals that are known to brominate exclusively. Finally, we were able to show that metagenomic data analyses constitutes a powerful tool to find new biocatalysts, for example for enzymatic halogenation. Metagenomic data has the great advantage that it not only provides genomic data of cultivable, but also of non-cultivable organisms. This expands the search field for novel enzymes. At the same time, the difficulty in determining the natural substrate of an enzyme from an organism that is not well-characterised remains a challenge.

## Supporting information

S1 Fig SDS-PAGE (12%) analysis performed after cell disruption of E. coli BL21_pGro7 with the overexpressed BrvH protein. M: prestained proteinladder, NEB (11–245 kDa); P: pellet with insoluble proteins; S: supernatant with soluble protein fraction; before induction: sample taken from E. coli BL21_pGro7 without induction with IPTG and L-arabinose. BrvH possess a mass of 56 kDa and chaperone GroEL of 60 kDa.
(TIF)

S2 Fig SDS-PAGE (12%) analysis performed after cell disruption and Co-IMAC purification by His-Tag of E. coli BL21_pGro7 with the overexpressed BrvH protein. 1–8: collected fractions after Co-TALON purification and elution with 300 mM imidazole. BrvH possess a mass of 56 kDa and chaperone GroEL of 60 kDa.
(TIF)

S3 Fig GC-MS analysis of the brominated indole product after 48 h of enzyme incubation. A: gas chromatogram; B: mass spectrum of the product from t_R_ = 20.79 min ([M+H]^+^ obs. 195.138 (^79^Br); 197.119 (^81^Br), calc. 194.968 (^79^Br); 196.968 (^81^Br).
(EPS)

S4 Fig ^13^C-NMR (500 MHz, DMSO-d_6_) spectrum of the brominated indole by BrvH. (EPS)

S5 Fig ^1^H-NMR (500 MHz, DMSO-d_6_) spectrum of the brominated indole by BrvH. (EPS)

S6 Fig ^1^H-NMR COSY (500 MHz, DMSO-d_6_) spectrum of the brominated indole by BrvH. (EPS)

S7 Fig HMQC (500 MHz, DMSO-d_6_) spectrum of the brominated indole by BrvH. (EPS)

S8 Fig RP-HPLC analysis of BrvH catalysed bromination of indole after 0 h, 24 h and 48 h at 280 nm. After 24 h of incubation the substrate, indole (t_R_ = 5.5 min), was fully converted to the halogenated indole (t_R_ = 6.1 min). Under these conditions a by-product is formed (t_R_ = 6.5 min, HPLC method B).
(EPS)

S9 Fig RP-HPLC analysis of BrvH catalysed chlorination of indole after 0 h, 24 h and 48 h at 280 nm. After 24 h of incubation the substrate, indole (t_R_ = 5.5 min), was converted in traces to the chlorinated indole (t_R_ = 6 min; HPLC method B).
(EPS)

S10 Fig RP-HPLC analysis of BrvH catalysed bromination of indole in presence of catalase after 0 h, 24 h and 48 h at 280 nm. Catalase was added to this enzyme assay for decomposition of hydrogen peroxide. After 24 h of incubation the substrate, indole (5.5 min), was fully converted to the halogenated indole (t_R_ = 6.1 min). A second by-product is catalysed (t_R_ = 6.5 min). These results show that free hydrogen peroxide is not responsible for the halogenation (HPLC method B).
(EPS)

S11 Fig RP-HPLC traces of indole incubated with BrvH_K83A and NaBr after 0 h, 24 h and 48 h at 280 nm. A point mutation at position 83 on amino acid level in mutant BrvH_k83A leads to an exchange of the conserved lysine residue to alanine. This experiment revealed that the conserved lysine residue is essential for the activity of the FHal BrvH. The mutated enzyme did not halogenate indole (1.2 min; HPLC method C).
(EPS)

S12 Fig UV/vis spectra of the halogenase BrvH after successful reconstitution with FAD in comparison to FAD in solution. Upon binding of FAD to BrvH, the band at 450 nm shows a slight shift to 448 nm and some fine structure while the band around 373 nm is shifted to around 360 nm. This latter shift is caused by the changed environment of FAD inside the protein compared to the polar and strongly hydrogen-bonded environment in solution, proofing that BrvH binds FAD.
(EPS)

S13 Fig Difference in the openness of the active site between BrvH und RebH. **A: Surface representation of BrvH.** The structurally conserved part between different halogenases („box“) is shown in dark blue; the structurally variable „pyramid”is shown in turquoise. B: Zoomed into the active site. The ε-amino group of lysin is shown in orange (centre of the image), showing its accessibility to the solvent in the apo form with no substrate bound. C: Trp (from RebH; shown as dark grey space-filling spheres) and RebH (dark grey, cartoon representation) are superimposed on the structure of BrvH to highlight the loop covering the active site in RebH. The RebH loop shown is the same as that shown in Fig 7A. D: Surface representation of RebH. The substrate binding site is not accessible to the solvent.
(EPS)

S14 Fig RP-HPLC analysis of the enzyme assay with all cofactors, compounds and indole, but without the halogenase BrvH for zero sample after 0 h, 24 h and 48 h at 280 nm. The substrate, indole (t_R_ = 5.5 min), is not converted after 48 h. This assay proves that the conversion of indole to bromoindole is BrvH (HPLC method B).
(EPS)

S1 Table Data collection and refinement statistics of the crystallisation of BrvH. (PDF)

S2 Table Metagenomic data, which were screened with the created HMM algorithm for FHals. (PDF)

S3 Table The 42 identified putative FHal genes from Botany Bay metagenome. The genes were identified by using our created HMM in a two-step approach based on the conserved regions of tryptophan halogenases.
(PDF)
