# Supplementary figures and images for: A flavin-dependent halogenase from metagenomic analysis prefers bromination over chlorination
Source: PLoS One. 2018 May 10;13(5):e0196797. doi: 10.1371/journal.pone.0196797 (PMC5945002; doi:10.1371/journal.pone.0196797)

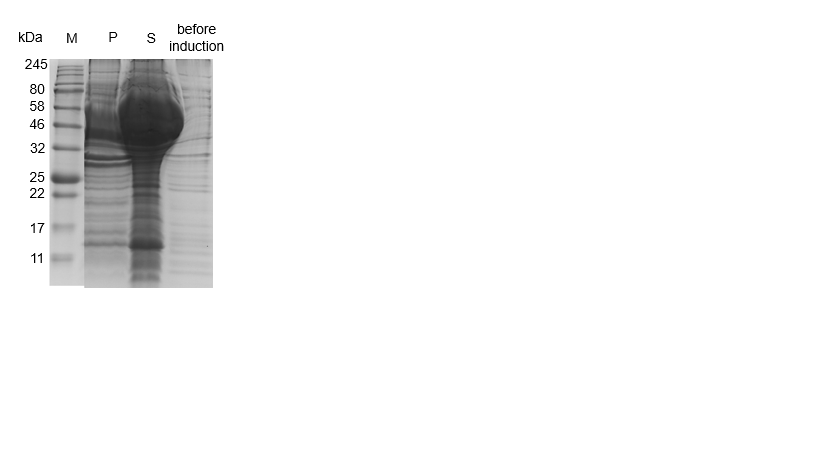

Supplement: S1 Fig — M: prestained proteinladder, NEB (11–245 kDa); P: pellet with insoluble proteins; S: supernatant with soluble protein fraction; before induction: sample taken from E. coli BL21_pGro7 without induction with IPTG and L-arabinose. BrvH possess a mass of 56 kDa and chaperone GroEL of 60 kDa. (TIF) [file pone.0196797.s001.tif]

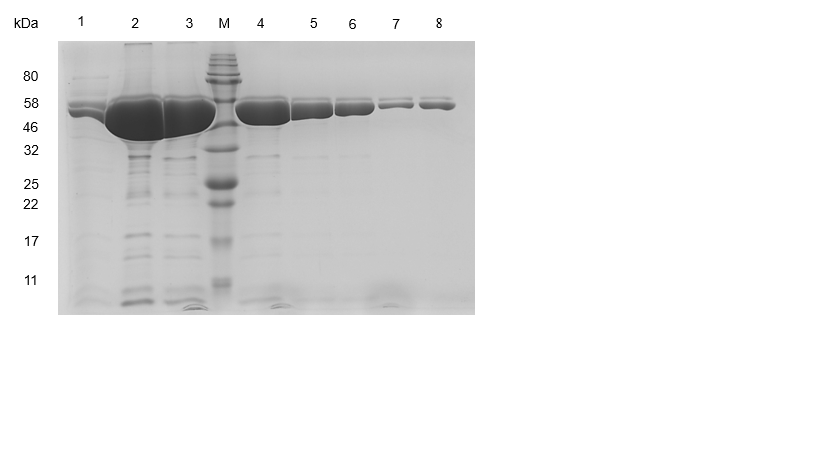

Supplement: S2 Fig — 1–8: collected fractions after Co-TALON purification and elution with 300 mM imidazole. BrvH possess a mass of 56 kDa and chaperone GroEL of 60 kDa. (TIF) [file pone.0196797.s002.tif]
