# Supplementary material for: A flavin-dependent halogenase from metagenomic analysis prefers bromination over chlorination
Source: PLoS One. 2018 May 10;13(5):e0196797. doi: 10.1371/journal.pone.0196797 (PMC5945002; doi:10.1371/journal.pone.0196797)
Supplement: S1 Table — (PDF) [file pone.0196797.s015.pdf]

S1 Table.

**Space group**	C2
**Unit-cell parameters (Å, °)**	180.62, 88.35, 70.11, 90.000, 99.075, 90.000
**Data-collection statistics**	
Wavelength (Å)	0.96770
Energy (eV)	12,812
Resolution range (Å)	49.32-2.5 (2.56-2.5)
No. of reflections	408829 (30741)
No. of unique reflections	36507 (2688)
Completeness (%)	96.5 (98.0)
R_{meas}	0.202 (1.422)
Redundancy	11.4 (11.2)
Mean $I/\sigma(I)$	11.89 (1.84)
$CC_{1/2}$	99.6* (66.1*)
**Refinement and model statistics**	
No. of reflections (working / test)	34697 / 1809
R_{cryst}	0.19456
R_{free}	0.24072
r.m.s.d. bonds (Å)	0.0128
r.m.s.d. angles (°)	1.5723
B factor (Å): Wilson / average	34.1 / 39.88
No. of non-hydrogen protein atoms	7732
No. of waters	315
**Ramachandran plot by MOLPROBITY**	
Favoured region	95.79 %
Allowed region	4.00 %
PDB code	6FRL
