# Supplementary material for: A flavin-dependent halogenase from metagenomic analysis prefers bromination over chlorination
Source: PLoS One. 2018 May 10;13(5):e0196797. doi: 10.1371/journal.pone.0196797 (PMC5945002; doi:10.1371/journal.pone.0196797)
Supplement: S2 Table — (PDF) [file pone.0196797.s016.pdf]

S2 Table.

Metagenome	Project ID<br>Gold ID	Collection Site	Habitat	File Size	Temperatur	Total hits	Complete genes
Kaneohe Bay	CAM_SMPL_000824 (www.imicrobe.us)	Kaneohe, Hawaii	Marine Sediment	40 Mbases	25°C	1	-
Botany Bay	CAM_SMPL_001699 (www.imicrobe.us)	Sydney, Australia	Marine Sediment	91 Mbases	25°C	42	42
Enigmatic life underneath	Gp0057578 (gold.jgi.doe.gov)	Juan de Fuca Ridge flank, Pacific ocean	Deep oceanic basalt-basalt hosted subsurface hydrothermal fluids, CORK Borehole	20 Gbases	Not specified	1	1
Perpetual spouter A (PS_A)MetaG	Gp0093345 (gold.jgi.doe.gov)	Yellowstone Nationalpark, Wyoming, USA	Perpetual spouter, thermal spring	16 Gbases	Not specified	0	-
Mammoth Hot Spring	Gp0054656 (gold.jgi.doe.gov)	Mammoth Hot Spring (Liberty Cap Streamers), Yellowstone National Park, Wyoming, USA	Environmental, Hot spring cone	6 Gbases	Not specified	0	
Tshipise hot spring metaG	Gp0138775 (gold.jgi.doe.gov)	Tshipise, Limpopo, South Africa	Hot spring microbial communities from South Africa	17 Gbases	42-90°C	0	

Sagole Hot spring metaG	Gp0138776 (gold.jgi.doe.gov)	Sagole Baobab, Limpopo, South Africa	Hot springs metagenome	14 Gbases	45°C	42	18
AOA Metagenome C0912_C49A8_80	Gp0056852 (gold.jgi.doe.gov)	Monterey Bay, California, USA	Marine metagenome, ammonia-oxidizing marine microbial communities	5 Gbases	Not specified	0	
Genome sequencing of two Panamanian Oscillatoria strains for drug discovery	Gp0054795 (gold.jgi.doe.gov)	Panama canal	Marine cyanobacterial communities from Panama	28 Gbases	Not specified	18	0
Metagenomic sequencing of an uncultivated bacterial phylum in marine sediments	Gp0112845 (gold.jgi.doe.gov)	Atlantic Ocean:Atlantic Coast	marine sediment metagenome	26 Gbases	Not specified	150	16
