# Supplementary material for: A flavin-dependent halogenase from metagenomic analysis prefers bromination over chlorination
Source: PLoS One. 2018 May 10;13(5):e0196797. doi: 10.1371/journal.pone.0196797 (PMC5945002; doi:10.1371/journal.pone.0196797)
Supplement: S3 Table — The genes were identified by using our created HMM in a two-step approach based on the conserved regions of tryptophan halogenases. (PDF) [file pone.0196797.s017.pdf]

S3 Table.

Predicted gene	Amino acid sequence
1	MAEEAYAPLKSIVIVGGGSAGWMTAAALSDAVGKSCSITLIESEAIGTVGVG<br>EATIPPIRQFNARIGIDEATFVKETAGSYKLGIQFVDWSRKGHSYFHPFGQY<br>GAEFDKVPFYHYWMREALDGRIAGPIDEFSMCWAMAKAGKFARPEPDRRK<br>IQSTFDYAYHFDAGLYAAFLRRVSEARGVKRIEGRVTDVIQRGEDGFIESVT<br>LDKGTSVSGELFIDCSGFRGLLIEDTLKAGYENWQHWLPCDRAVAVPCAKQ<br>AEITPYTRSTAKEAGWQWRIPLQHRTGNGYVHCSEFISEDEATETLMGSLD<br>GEAQADPRTLRFVTGRRKKFWDKNCVAIGLSAGFMEPLESTSLHLIQYGILR<br>LIALLPDSEMSPLLAREYNRQTADEYARIRDFLILHYKATTRDDAELWRYCSA<br>MDIPESLQYKIDHFRNYGLLVADERELFANPSWIAVYMGQDIMPRRAPAITE<br>MRDGVPVAQRMQMISQAMREAVAQMPTHADFLARHCPSPLAS
2	MSNTNVQKLVIVGGGTAGWITAAAFSRLMGRTLSIELVESDAIGTVGVGEAT<br>IPQIIRLNRILGLDEHDFVKRTNGTFKLGIEFVDWGDVGTRYLHTFGDAGMNL<br>ASVPFHHYWKRAEASGSRHSLWHYSLHQLAANHAKFGKLDRVGNTSMTG<br>LAYAYHFDASLYAAYLRAFSEQKGVTRTEGIVQSVARDGESGDITSITLDNG<br>REIAGDFFIDCTGFRALLLGQELGVGYQDWSKWLPCDSAQAVPSERLETLV<br>PFTRATAKTAGWQWRIPLQHRTGNGHVYCSDFISDEEAGETLLAGLDTKPL<br>ADPRPIRFTTGRREQFWSNNCAAIGLSSGFLEPLESTSIHLIQSHVSRLLQLF<br>PASNRSDVERAEYNRRCEAEFEQIRDFLILHYHQTNREDSEFWRYCRNMD<br>VPDSLTHKQELFASSGRVGRDADDLFREASWVQVMLGQGVTPQSHDAMA<br>NGLSDAQLSEFLGNVRTLIERAVGTLPTHEQYLKQNCAAAEAA
3	MAIQHIVIVGGGTAGWMAAAALSRLKSGRTLDITLIESEQIGTVGVGEATIPP<br>FVGFNDLLGIPESELLSNVQGTFKLGIQFENWGKRGDSYIHPFGAYGYTMG<br>GISFHHVWHKLAKEGDNRPIQVFNVETMAAYFGKFMRSEDYQKQRDDLPPI<br>NYAYHLDAGKYAAFLRGYAEKRGVVRQEGRVDDVSLDGETGFVTGVTLDD<br>GRKIDGDLFIDCSGFRGLLIEQALGTGYEDWTHWLPCNRAVALPCGRDDGS<br>PPPPFTRATAHSAGWQWQVPLQHRNGNGHVYCSEFMEDQEALDILLGNIA<br>GKPQADPNFLRFTTGRRKKFWNKNVVALGLSAGFMEPLESTSIHLINTGVD<br>KLLSLLSLDGVTQTQEDTFNRLTGREYARIRDFLILHYNATSRDDSGFWNYV<br>RTMDVPETLTEKVELFKANGQIFREEDELFTETSWAAVMMGQGIAMTGHNA<br>MADSLDPASTRKEIDEMEQSIRFLIQHMPGHGEYLSRYCPAKAA
4	MTQPSLPRRIVVAGGGTAGWMTAAALARTLGPVAQVTLIESEQIGTIGVGES<br>TIPPLVAYNRILGIDEADFMRATQATFKLGIQFENWRVPGESYFHSFGGTGK<br>DHWSAGFQHFWMHGAAQGHDAPYDEYCLELKAAMAGRFAHLPDDRMNY<br>AYHLDSGLYAKFLRAMAEGDGATRIEGRIVAVDLDGASGDIAALRLDGDRRI<br>EGDLFIDCTGFRALLIEGALHAGYDDWTHWLPCDGAVAVQTASVRAPVPYT<br>RAIAHDAGWQWRIPLQHRQGNGIVYCSAFMEPQAAQDQLLATVEGDRLVQ<br>PNPIRFRTGARRRQWHRNCVAVGLSGGFMEPLESTSIHLIQRAVLRILRMLP<br>AGAISPRDIAEFNDQQMADMEQIRDFLILHYKATDRRDTPFWRHCASMSVP<br>DSLTHKMELFRETGRVFRKNEELFAENSWVQVMMGQGITPQSYHPVAAKL<br>RPQELKHLLTALRDQVSRTVSALPDHGAYVARYCGAQDVAAA
5	MVRPVRNIVIVGGGTAGWLTAGVIAARHRTRIGQDLSVTLVESPNTPIIGVGE<br>GTWPTLRTTLSRMGISETDLFRQCDAAFKQGAWFARWTDGTASDGYYHPL<br>MLPQNFGQVNLVPHWLARDGDESFCDAVCAQGRICDDGLAPKTMATAEY<br>DALANYAYHLDAGKFAPFLQRHCCEKLGVRHVLADVEQVVLHDDGDIAAVR<br>TTQAGLVEGDLFVDCTGFKALLIEGAMGVPFRDCNDVLFCDTALAMQVPYA<br>APDSPIASHTISTAQSAGWIWDIGIQTRRGVGHVFSSSHISDEQAERELRAY<br>VGPASDGLNVRKIKIRSGHRETFWKGNCVAVGLAAGFLEPLEASAIVLIELSA<br>KIIAEQMPACREVMDIIASRINATTHYRWGRIIDFLKLHYVLTQRTDSDFWRD<br>NVRAETMPDRLRELLHLWRYQSPWFHDEFDRAEEVFPAASYQYVLYGMG<br>FRTQVPTAALARDAHLAGRAMRENAAATARLRAGLPRHRDLIDRIVAQGLQ<br>PV

6	MTKTNAVKNVVIAGGGTAGWMAAAALAKLIGKNIHVTLVESDDIGTVGVGEA TIPPIRTFHKLLGINEQAFMKATQASFKLGISFENWGDENTRYIHSFGVTGKE CWAGEFHHFWLQGKVLGINNPFGDYCYELQAAKAGKFAFNQQNPINYAYH MDATRYAQFLREFSEPLGVKRVEGKIQKVVKSAKTGHIESLLLHSGEQINGD FFIDCTGFRGLLIEQALHTGYEDWSHLLPCNSAIAVQTKSTSDTVLPLTRSIA HKSGWQWRIPLQNRVGNGLVYCSQYMDDEQAKALLLNNIEGEALTEPRVIK FKTGRRLKGWNKNCLALGLASGFVEPLESTSIHLIVSGIIRFMRLFPFNGITN YAIDEYNKQLKSEIENIRDFIVLHYKVTNRDDSEFWQHCSAMEVPTTLQHKID LFKQTGRVFLDDGDIFRVDSWVQVMLGQGIVPEQHHLIASMMSDEELARFL HGIKQQIEQRVSQLPPHHEFLKHYCPA
7	MNKAVKTVAVIGGGTAGWLSAALIASYHQSQNGDGLKVMLVESSDIPTIGV GEGTWPTMKNTLQQIGLREVDVFKTCNATFKQGGKFVNWTHGNNDFYYH PFTVPLGYGRIDLAPYLDNVVDYAQQTNFQHDICEASLAPRGIAQGEYQGN CNYAYHLDAGAFAEMLKQHSKSKLGVEHVIGTVDEVKLDNDGAIDTLTLKD QTRTINADLYVDCTGFSSLLLGQALNIPFKKMDHVLFNDSALAMQVPYAEGD ESLASHTIATAQNAGWIWDIGLTHRRGVGHVYSSKFLSDDEAEQNLRNYLG DAAEGLTARKISFESGYREKFWHKNCVAVGMSAGFVEPLEATAIMLVEISS RFVAEHMPADTKVMPIVAKRFNEQMDYRWQRIIDFLKLHYMLTKRPEPYWQ AHVDPDTIPQSLKDDLLLWESRGPLIQDFHGALELFPAASYQYVLYGMGFK PDFTKQAYLYSQDAQAKQIIERNSQLTQQMLQTLPPHRAYIEQWLAANPV
8	MNQQNKKIKAVVVVGGGIAGWLTAGRLAAHHKSNTPDGLNVVLVESPNVPII GVGEGTWPTMRSTLIALGISETDFIRECEASFKQGAKFAKWVDGSEDDFYY HPLVLPQGFSKTDMAGHWLNNTAESTSFSNAVCYQEAICEQGLAPKTIRTP EYAAIANYAYHLDSAKFAVFLQKHCTQQLGVKHILADVTQVNSADNGDIASVI TKQQGEISGDLFVDCTGFSSLLLGEHYKVPFMPCDDVLFIDTALAVQVPYEN DDENIASHTISTAQEAGWIWDIGLQHRRGVGYVYSSKYSSEAQAREKLKAY VGDKIESLTIRKIPIKSGHRSHFWQNNCVAVGLSAGFLEPLEASALVLVEMSA QMISEQLPQTRDIMDITAKRFNETFTYRWEKIIDFLKLHYILSTRDDNAFWRD NRDPKTIPQSLQDLMKLWQYRAPADHDFTSNNEVFPAASYQYVLYGMGFK SDYSTTTYMLKDTVFAKSQFDKNKSMINQAVTNLPSNRELLNKIKHHGFSAI
9	MTKPIKKIVILGGGTAGWLSAGLLAAEHPHLSVTLIESANVPILGVGEGTWPS MRNTLQRIGIKEIDFITQCDATFKQGSKFINWCNLSKGENYYHPFMDPQGYE QTNLHANWQRIAPNQKFADVVNMQSFVCQAALAPKQLQTPEYAAVTNYGY HLDAGKFAEFLKNHCVKHLNVTHNTDDVTHVINDEHGYIASLKTNKNGDITG DLFLDCSGSRARLIGEHFNSEFIAQDHILFNNSAVAAQVPNAHINQPIASATL STAQSNGWIWDIALPTRRGTGYVFSSKYQSGESATDTLKSYIAKSTSEQQA EQLNYRVLKFSPGYRQKFWIKNCVAVGMSAGFLEPLEASALAMVELSISML SEQLPQNRTHMSQLANRFNDRFSTRWQRVVEFLKLHYVLSERDDTPYWQ NIRDLTTAPEHLQNLLKLWQFQPPSRFDFIENEEVFSSSSYQYILYGMGFNT QTPQAHNAFTSAQAGDYFYQQNREKIAQYLKSLPSNRALLNDLLSNNRQPH ND
10	MKPIKNVVIAGGGTAGWIAAALLNKVLGKVINITLIESSSIGTIGVGEATIPPIIQ LNNALGINEQDFINATNASIKLGIEFENWKTPSHSYMHAFGSFGKDFPFCDF YNFWVKGQISGSEDSLWDFSLNYQAAKKHKFAPLSAIPNTQLPGISYAFHFD ATLYAEYLKKLAVSRGVKHIDAKIERVTQCPNTGQINSLTLDNNSQIEGDLFI DCTGQRALLIEQTLNTGFVDWSHYLPCDSAVAVQSTGSDELRPYTRSIAHN AGWQWQIPLQNRTGNGLVYCSRYLSDEGATALLLNNLPGEPTTDPRLIKFK TGRRLKQWHKNVVAVGLSSGFLEPLESTSIHLIQSAVIRLIKLFPHNGISDALV TEFNKQSVTEIEHIRDFIILHYKLTEREDSPFWRQCKQMDIPQSLAHKIDLFR QTGKVVRENDELFAEVAWQQVCIGQGLIPEDYNNIVESLSSEQLNDLFSTLK TLITSTVDQLPTHKDFLAKIKKA
11	MTAAALARLVAPSGVQITLVESEAIGTVGVGESTLPHIRAFNERLGIDEAAFM KATRATFKLGIQFENWGQIGGSYIHPFGDYGAPGGEAPFHHYWLRGRELG DVGPIDDYSLPVVAAREGRFAPPSDDPRSLLSTYRYAYQFDATLYARFLRG MAETEGVRRIEGRIQNVAQDGESGAITKVTLDDGRQIEGDLFVDCSGFRALL IEQTLKAGFTDWSHWLPCDRAVALPCESDQPTVPYTRATALEAGWQFRIPL

	QHRVGNGYVYSSAFIDDAAALDRLVGRLEGKPLKAPNVLKFTAGQRTRNW AANCVSVGLSSGFIEPLESTSIYLIQIAITKLIELWPTAVIDPATEAEFNRQM DLEYIRIRDFIILHYCATARNDTPFWDYVRTMPLPDTLSEKIELFRARGVVQD YREGLFLHPSWVAVYLGQDILPRRHHPLVDAVSATQLMSSLERLRRMVREA SYKLPSQDDYIARYCAHLPL
12/*brvH*	MDEIDDPRIRSVVIVGGGTAGWMTAAALVQHFRTAPLKITVVESSDIGTIGVG EATIPTIRRFYGQLGLRDDDVMRATQATCKLGIRFLDWSGPGSDFIHPFGLY GQDVKGIGFHHYWLKQRRAGDAAPLAAYSLGAALAAGGKFTLPSPHPPSQ LSVFDWALHLDAGLFAQHLRAYAEAGGCARIDARIRSVELRPEDGFVRALTL DDGREVEGDLFVDCSGFKGLVIGEALGVGFEDWGRWLPCDAAYAVQSEN RPGDAPAPFTRVTARSAGWQWGIPLRHRAGNGLVFSSAHLSDDQALAELM PHLLGDPLTEPRRIPFRPGRRSQAWAKNCVAIGLSSGFLEPLESTSIALIETG IERLKALFPDRRFAQPILDEFNDQTAREMERVRDFIILHYKLNRRTDTDFWR DCREMPVPETLERKIALWTARGQFVRYRWEMFHPASWLAIYDGFGLYPDH HDPAVDAMDPAYLARSLAEMRANIADLVARTPEHAQFLAGLDPAASAA
13	MDQPRRPLKKICIVGGGTAGWVAAALMAEHFKGRMAEIELVESDDIGTIGV GESTVPPFLEMLKRLGIDERDFIQKTQASFKLGIQFEDWTQKGESYFHPFGA VGVPVDISDFYPVWLKARMNGYDRPLMDFAAAARMASQGRFMLPFKAQRT PIGGASYAVHVDAKRVARYLRDFAEARGVKRTEGIVEDVMTRPDGFVSGLK LKSGQTVEADFFIDCTGFRALLIGKTLGVGYTDWSEWLMCDRAIAVQTENV GPPTPYTLAQAQDCGWRWRIPLQHRTGNGHVFCSRYMSDDEATARLLAQ VEGRPVVNPMVVPFKTGVREKIWDKNVLSLGLASGFIEPLESTAIHLIYRGM DFLFRLMPDLDCDPNLAAEYNRRMTVDYEEIRDFIILHYALTRRDDTAFWRQ CRDMVLPEGLQHKLEVFRANGSLVEALDPLFRNVSWYAVMDGMGVRPRS YPPLVDRIDSTGLTDEMDRAANILNAFVADLPTHQAFIDTHCPAPSVDLPAPL AVPA
14	MGADVRQLKTILIVGGGSAGWMAAALFSKLFKGLYDITLIESEAIGTIGVGEA TIPAIKTFNELIGLDENDFMRRTQASFKLGIQFVDWDRVGSSYIHGFGVIGRD LKWLRCHQYWLKMRAEGKVSDLAAYSINTAAALQNRFMRADPKRPGSPLG HIAHAFHFDAGLYAKYLRGMSEAAGVRRREGKVVSVQLRPEDGFVDSVTLE DGAVVSADLFIDCSGFRGLIIEQAMKTGYEDWSHWLPCDRAMAVPCARTEP FTPYTRSTARPAGWQWRIPLQHRTGNGHVYSSRHMDDGEAEQILLSNLDG AQMAEPNRIRFVAGKRRQIWNRNCVAAGLASGFLEPLESTSLHLIQSVLIRL VRLMPDAGFDPATIAEFNRQTDFEYERIRDFIILHYKATERDDTPFWRYCRD MDVPVTLERKIDLFRANGRIFREDDELFAEESWIQVLLGQGIIPVGYDPLVDV TPEPDIERYLADVETVIGKCVALMPSHADYVAKACPAA
15	MAAAALSKLIGKQLDIALVESDDIPTVGVGEATIPTILTINRLLQIPEPEFLQAT QGTFKLGIEFENWRKQGHRYIHSFGDTGQGCWAAGFHHFWLKGMEQGIS SDYGEYCPELKAAEQSKFAIQSQKKLNYAYHLDAGLYGRFLRKIAEQHGVR RIEGKISDVQCNTDDGYIQSLRLESGQVVEGDLFIDCTGFRALLIEQALESGF DDWSHWLPCDSALAVQTESVGEAVPYTRSIARCSGWQWRIPLQHRVGNG FVYSSKYLSDDKAEQILLDNVEGAPITKPRPIKFRTGQRKQYWNKNCIALGL SSGFIEPLESTSIHLIQNGIMWLLLMFPFQGITKSVVNEYNEKLRREADCIR DFIILHYHVTERDDTDFWNYCREMSIPDSLQHRIDLFRETGRVFKPQDDVFA ENSWVQVMMGQGITPEQYHPIVDMMSDVELKSFLQTIENNAKSLVSQLPSH ADFVAHYCKAVG
16	MVNRIEKVVVVGGGSAGWLTAATLAAEFKSGPGGISVTLIESAETKSIGVGE GTWPSMRTTLQKIGISETEFFRECDASFKQGSKFTSWLDGGNESYYHPFSL PEGYQEINLADFWAPHRDKVSFANAVCPQLRVCESHKAPKQISAPDYAGSL NYGYHLDAIKFARLLRKHAVEKLGVTHVIDHVEKIHSAPNGDIASLQGKAHG QINGDLFIDCTGFASLLIGRHYSIPLIGKKDVLFNDRAIAVQVPYADESAPIAS ATLSTARSAGWIWDIGLPSRRGTGYVFSSKYIDEQQAVDELKAYVGNSAGIP FEELAPRTIDINPGYRQKFWHKNCVAIGLSAGFVEPLEASALVLIELSARMI AEQMPADRQVMDIVAEKFNDKFATRWQQIIGFLKLHYVLSRRNDSQYWLD NRSAESIPSDLAAQLKLWRTRSPWYCDETHVDQMFPSASYQYVLYGMEYP

	SQPMTARKRSGDSDQQRARQIFASNDSMAGKLLTNLPTNRDLTDQVKHQG FGHI
17	MLGGGSSGYFAALAIQRRFPELEVTVIASSKVPIIGVGEATTTLMPPFLHAQL GLSMEAMCRAVEPTFKLGIRFEWGRPGAYHFNFPFGDADPLLAHHFDGDL RHQSLMSMAMDQGLAPVLRGPEGEVVSLLRQVKFAYHLDNAPFVAFLAEA ARAAGIGHIDATICDASTRAGASGEPEIASLRTEDGRELAYDLYIDASGFRSV LLGGALEERFQSYASSLFCDRAVVADVAEGAGVEPYTTAETMDAGWCWRI PVRRACHRGYVYSSAFQSDDEAVAHMRAANPSMGEAKLIRFRSGRRERA WVGNVAAVGNAYGFVEPPRVHGPAHGHRRARLRARGDRGPASRGRGRG RGADLPRVRQRVPGRALGLPALVSGGALQVQPAPGQRLLAGRPGRGRRL GPRAGDRALSEPGAVAGRGRPGLRRGRSSLRLQRADDHAARPARREPGR CGAQNQAEPGPVARADRASAHRPALRRGSAHGLGLARGPPRAPARVRGA PQLVVPGRRRAGRGDRRGSAAGTAQERHARGASRLAASGWGLARGPRP DPRMGERLTRA
18	MRMAIEHIVIVGGGTAGWMAAAALSRIRDGRDLKITLVESEQIGTVGVGEATI PPFVEFQNALDIDEREMMAAVQGTFKLGIQFANWGQVGESYIHPFGDYGYE LGGVHFHQVWHAMRERGDKRPLQVFNLETMAAHFGKFMRSQDYGRDDLP PMNYAYHIDATAYARFLRAYAEKRGVVRREGLVKNVALHPETGFVTGITLDD GEEIEGDFFVDCSGFRGLLIEQALETGYEEWTRFLPCDRAVALPCNRDDGS PPPPFTKATAHKAGWQWQVPLQTRNGNGHVYCSEFMEADEAHDILVQNLA GKPTADPNHLRFVTGRRKKFWNKNVVALGLASGFMEPLESTSIHLINTGVN KLIATLSLDGVTEAQASTYNRLTDKEYERIRDFLVLHYKVTRRDDSEFWNYV RTMDVPDTLSEKIEIFKANGQVFREEDELFTTTSWAAVMLGQGITMDGTSP MAAGVIDGVAKEVDEMERSIKWLVQQMPGHDQYLQRYCPTGVKAA
19	MQDPHNKPIKRVVIAGGGTAGWLAASLMKKVLGKAVDITLVESEAIGTVGVG EATIPPIRLVNQVLGIDEAQFLHDTKATIKLAIRFENWKTKGESYYHTFGAPG KSMAFCHFHHYWVKARQQGLKADLWDFDLNYHAAEAGRFAQINAKDPVVE LPFAYHFDASLYAQYLRKLSENMGVVRKEGKISRVQRFTDSGFIQALHLESG DVVEGDLFVDCTGFKGLLIEEALGAGYDDWSHLLPCDSAIAVPSERHEKTA PYTRSIAHDAGWQWRIPLQHRNGNGHVYSSRYISDDQACDTLLSNLDTKPL GDPKLIKFTTGRRRKQWYKNVVAVGLSSGFLEPLESTSIHLIQSAIVRLIQLFP HNGFAPSLESEYNKQSELEFEQIRDFLVLHYTVNERTDSAFFNDMRNITLPD SLAHKIALFKESGNLLREQNDLFLESSWLQVLYGQGITPKDYHGLVNSVPEV QLNQMLTRLLEIKKEPIAKLPTHDEYINGMVAKYKRAMS
20	MNNRLRKITIVGGGTAGWMTALILETELSRTSAPKDRPKICLIESPNIATVGV GEATVPRMPKTLRQAGISERAFFRETNASFKLGVKFCNWNKDANGNRIDYV NPFAHGQLLDGLEPAEYFLRFGNGDRDYTQSIAPHDDLGRLCKGARPLGQ PEFEQRFGYAYHLDAVKFAGMLTKVCTKHGVEHIQDEVTSVELDEQGNVS HLMLEQRGRHDIEMVIDCTGFRGLIINQALGEPFMDYSDYLPNDRAMALQID HPDPEKIESLTRSTALGAGWTWRVPLYNRVGTGYVFSSAHRTDDQAADEY LEWLGDSGKGATPRVIPMRIGRVRNAWVKNCVAIGLAGGFIEPLESTAIHM IDHAVRWLTEHMPTRDIAPSLRTRYNRQMDKLYDEVLEFICLHYRLGNRTD DQYWIDARTEMKIPDRLAENLELWQHRLPMPHDIEFATLFDYRVYQTVLLG KQVYDTGYGPGIRDRLRPLKKPIWFQWWKGAKVDLANILKSMPDHKTLLRD IRGELDQPGFGMGAAMKPTVSMPCPEAVPCMIQNMPSFSEIENGQKDLQL F
21	MDNRLKKITIVGGGTAGWMTALILETEFARTSAAKDRPKICLIESPNIATVGV GEATVPRMPKTLRQAGISERAFFRETNASFKLGVKFCNWNKDAKGNRIDYV NPFAHGQLLEGLEAAEYFLRFGNGDRDFTQSIAPHDDLGRLCKGARPLGQP EFEQRFGYAYHLDAVKFAGMLTKVCTKQGVEHIRDEVTSVELDEHGNVSHL MLEKRGRHDIEMVIDCTGFRGLIINKALGEPFLDYSEYLPNDRAMALQIEHPD PEKIESLTRSTALGAGWTWRVPLYNRVGTGYVFSSAHRTDDQAADEYLEW LGDSGKGATPRVIPMRIGRVRNAWVKNCLAIGLAGGFIEPLESTAIHMID HAVRWFAEHLPTKEIAPSLRSRYNRQMDKLYNEVLEFICLHYRLGNRTDDQ YWIDARTEMKVPDRLAENLELWQHRLPMPHDIEFATLFDYRVYQTVLLGKQ

	VYDTGYGAGIRDRLRPLKKPIWFQWWKGAKMDLAQILKAMPDHKTLLRDIR<br>GELDQSGFGIAAAMKPTVSMPGEAMAPFAVQNMPSHTEIQTGSKDLQLF
22	MMKVVIAGGGTAGWMAACGLSSRLASLLDITLIESDEIGTVGVGEATIPPMR<br>NFHRLMGIDEQEFMRETQATFKLGIEFDNWGNIGDSYIHSFGEIGQRSWMA<br>EFHEFWMEAQAQGFGGSLEDYCLELKAAKAGKFATAIGDTRLNFAFHLDAT<br>RYAKYLRKKSEKAGVRRVEGLIEDVQKDPQSGEIKALLLKSGELIEGDLFIDC<br>TGFRSLLLGESLGVEFDDWSHWLFSDRAIAVQTESIDEPVPYTRATAHPSG<br>WQWRIPLQSRVGNGIVYSSRFMSDDEAKQTLLNNISGKMITEPRHIKYRTG<br>RRQKSWHKNCVALGLASGFIEPLESTSIHLVMTAIIRLIRLFPFGDSSTALAD<br>RFNQESTTEIETVRDFVILHYKQTNRTDTDFWNAYRTMDIPDTLAHRLDIFKN<br>NGYVWPDDVALFRVDSWVQVMMGQGLTPSGFHSAGKLTGSEGLQQSLAK<br>LKTSIDEKVAKLPTHQQFIDHYCAAPKAD
23	MTKTVVIVGGGSAGWLTAGIIAARHNPLGEAGQGTKVILLESPDVANLGVGE<br>GTWPTMRDTLRRIGISEGDFLRACDVGFKQGSQFINWQRGEGESYYHPFV<br>PPAGYGSVNLAAHWLDKAVDSTFADAVSPQGKVCDLGLAPKTARTPEYAF<br>GLNYGYHLNAGKFVELLKRHCVDKLGVDYQLGHVDQILSDENGDISALLLTA<br>GDKISGDLFVDCTGFSALLIGQHYQVPFVSQRDCLFNDTALAVQADHADED<br>TPIASCTKSTAQSSGWVWDIALPTRRGIGHVFSSQHISVEKAEDELQRYLAA<br>DPALAKKQHSPRKITFQPGHREHFWHKNCVAVGVSAGFVEPLEASALVLIE<br>KSAEFVSNNLPQDRGAMTVVAKRFNEIISKYWVDIIDFLKLHYVLTERTDSDY<br>WRDHVRPQSIPDSLQDSLELWRSQVPWVSDGNNRAELFSAASVQYILYGM<br>GFATQINSSRYRDWDKDALLADRLMRDNRGKTEALMASQPTNRDLLNLVR<br>RTS
24	MNHKINRVVIVGGGTAGWLSAGIIAAEHCIGTKTSSPENPFQLTLVESPDIATI<br>GVGEGTWPSMRSTLQKMGISETDFFRECSASFKQGTLFKGWRTGADDIYT<br>HPFTPPQGYTEVNLAPHWQTAREQINFAQAVSPQSALFTDKLAPKQISTPE<br>YAFNVNYGYHLDAAKFAEFLRKHCVEKLGVTHIKANVTGINSADNGDIASIAT<br>DSHGTIEGELFIDCSGSRALLIGEHFGIPVCSKSDILFNNTALAAQIPYTHEND<br>PIESCTLSTAQSAGWIWEIGLPTRRGIGHVYSSDHNTEERASEELLAYIASTV<br>SDQAAAAATLRKVKFSPGHRAKFWHKNCVAIGMSAGFIEPLEASALVLV<br>ELSAAMIAEQLPANRQIMDLVAKRFNDKFLYRWDRIIEFLKLHYILTQRRDTE<br>YWRDNCESSSVPEQLVDLLALWRHQSPWHRDNNNVDDMFPSASFQYVIY<br>GMDFETMPSQTQRRSELAERQRAGALFQENATRTQQLQTSLPSNRDLINKI<br>YTFGFQKI
25	MTDKNITKVVVVGGGTAGWMAAASLSKLIGPNIRVCVVESDEIGTVGVGEA<br>TIPTMMTLHQLLKIDQPEFMAAVQGTFKLGINFENWNKLGDSYMHAFGRTG<br>QDCWAGGFQHFWTRGKELGISEGYGRYSASYVAAKAGKFAVLPDHSLSYA<br>FHMNATLYAKFLRNMAEKHGCERVEGKITQVNTHSDSGYIDSITLESGQKIE<br>GDLFVDCSGFRGLLIDETLQTEYEDWSNWLPCDSAVAVQTESVGEPIPYTR<br>AIARDAGWQWRIPLQSRVGNGMVFCSKYLSDDEAVDSLLKNVEGTPLTKP<br>RIIKFRTGQRKQHWNKNCIALGLASGFVEPLESTSIHLIQRGIIRLLQMFP<br>HQGICQPDVVEFNRQMSDEFVFVRDFIVMHYHLTERDDTPFWRHCQSMPI<br>PDGLRHRLDLFKQTGRVFQEAGDVFAENNWVQVMLGQGLLPEQYHPIVDS<br>MSEAELDNFLKGLAGNIDHLVNQLPSHQSFVSNYCPADSV
26	MEHQKIKHVVIAGGGTAGWMAAAAVAKLLGKTVKVSLVESEEIGTVGVGEA<br>TIPTLLTLHELLKIKEQDFISAVGGTFKLGISFENWHDVGKDYIHSFGYTGKD<br>CWAAGFQHFWLKGKELGISKDFGEYCNEWAAAKQNRFAVLPNQNLNYAY<br>HFDSSRYATFLRKIAEEHGAVRIEGKIEAVQQHEQTGFITGLRLESGQQVDG<br>DFFVDCTGFRGLLIEQTLHAGYDDWQHWLPCDSALAVQTENVQPPIPYTRS<br>IAHEAGWQWRIPLQHRTGNGLVYCSKFWSRDEAEAKLRGNLEGELVTDPR<br>PIKFQTGTRRRHWVKNCVAVGLSSGFMEPLESTSIHLIQRSIVRFMQMF<br>PSDGVRQPDIEEFNNQMKFEIENIRDFIILHYHVTNRTDTPFWRHCRTMEIPE<br>SLQHRIDLFKETGRVFKVPTELFGENSWTQVMLGQGLSPEQYHPIVNMMD<br>EQELRDFLGGIHGSVERLVSQLPEHQRFIDHYCREA
27	MGTTEHVVIVGGGTAGWLTAARLGAMANRQFDITLVESPSVPTVGVGEGT<br>WPSMKATLQAIGLSERVLISECDASLKQGTLFHGWRTGAADDSYLHPFSLP

	PEYASKNIAEYWRRDGLRYPFHEVVTPQALIATTHKAPKTADTPDYAFALNY<br>GYHVDAVKFAALLRQHSVSRFGVRHIEGHVAGVSSDSAGFLTSLELEGGDR<br>LSGDFFVDCSGQKALLIGDHFKVPFESARHVLPNNRAVVAQVPYNEPNDEI<br>HSCTQSSAQDCGWIWDIGLQSRRGVGYVHNADFISEAEATQTLRNYAEQS<br>VGAKVAQDLACRTLNFEPGYRGTPWTHNCVAVGLSAGFIEPLEASALAM<br>IEQAASFLVESFPVNRDLMGPASRAFNRKMESNWASIQEFLKLHYVLSDRD<br>DTPYWQQACDETSISEGLSDKLALWQIRAPWHPDTPRIDELFPAASYQYVW<br>LGMNGHLNGVIGSAGQGGKDHASSSADLDPILFKVRERALQLSRAMPGNR<br>QLLNALRMNNPSATVTA
28	MRVVIAGGGTAGWMAAAALAKYFGGTVAITLVESDEIGTVGVGEATIPQIRL<br>FNSGLGIDEREFLRETNGTIKLGIEFVDWRRRGERYFHGFGEVGRAIGLVPF<br>HHYWLRYAGEGGELPLEEFSPNAVAAREGRFGPGAGVAGARLPASAYHFD<br>ATLYARFLRRYAEARGVTRREGKVLSCTRNGGTGFIEALEMEKGDRIAGDL<br>FIDCTGFRALLIGETLGIDYEDWSHWLPCDRAFALPSEGRGRLDPFTRSTAR<br>GAGWQWEIPLQHRTGNGHVFASAFTDEQAALATLTGTLPGKALAEPRLLRF<br>ATGKRRRMWHGNCVALGLSSGFLEPLESTSIHLIQAGIARLLALFPDRSCD<br>PALVEEFNRQLDFEYEAIRDFLILHYHATDREDTEFWRYCRTMAIPESLSDK<br>MELFRASGRIVRFNTELFDVPSWLQVMWGQGLRPRTYHPMVDAAPDADLT<br>RYIAMNAGEVRQQVDTLDLHETYIARVARAGRDTAAPAGGG
29	MRVVILGGGTAGWMAAAALSRYFGDRLSVTLVESDAIGTVGVGEATIPQLR<br>LFHEGLGIDEADFLRATGGTIKLGIEFIGWQREGTRYFHGFGDVGRKIALAPF<br>HQYWLRYNAEGGELPLEAFSVNAEAARRGLYGVRPGAAAARLPASAYHFD<br>ATRYAAFLRRFAEAHGVVRREGRVRAPELTASGDIAALVLESGARLSGDLFL<br>DCSGFRALLIEEALGAGWEDWSHWLPCDRAL<br>AVASAGDGRLDPFTRASARAAGWQWRIPLQHRTGNGHVYASAFTTDDAA<br>HDALMTTLPGEAQGESRPLRFTTGRRRAFWRRNCIALGLASGFLEPLESTSI<br>YLVQSGIARLLALFPPPGGDATALRAQYNRQMAFDFALTRDFLILHYHLTER<br>PGALWEHCRTMALPDSLAEKIALFAERGEIVRFNGELFDVPSWLQVMWGQ<br>GLRPARHHPLVDAARPADLARYIDDTARDVAARLAPLDSHAAFIARRAAAPA<br>PAL
30	MTGPARHIVIVGGGTAGWLAALILARRDDLRVTVVESSRIPTIGVGEGTTAVF<br>RQMLQHLGLDERAFLAATGATIKYGIRHRDWRRVGHSYDGPIDDTAQVAGP<br>GLDLYAVASGRPVAEVHLFGTLMDRRRAPVAEVGGREVPAGPFHWAYHFD<br>QARAGAWLKRQATGVTVVDDVVAGLERDGEGGDITALRLESGARLEGDLFI<br>DCTGFRRALIGPMGAEWVSYGDVLPVNRAMPFWIDLQEGEEIDPFTLAHAQ<br>GAGWMWKIPTTERYGCGYVYSDAHTTPDAAQAEIEAALGRAIEPRNDIRIDA<br>GRLREAWIGNCVAIGLASSFLEPLEATSIHGTIVQLMWLDSLLAAPDGAARY<br>NAAVARQVDDFRDFIRLHYVSERRDTAFWRDVAASHPPEVRARLDLWRHR<br>LPERRDFAPVPLDLPHVQEQLYTPVLDGLGLLDRASARAALADHPHRRARL<br>RKTHEQLTKDHRRAAGRCRPHRAWLSSLREEIPA
31	MGQPLSSITIVGGGTAGWLAAVYLRRACAERIRITLIESPNIPTVGVGEATVP<br>QMPFTLRQIGLDERTFFRRCDATFKTGVMFRNWNTDRKGKLVSYLNPFSN<br>APSLDGVSIGNHFHAFGAGKRDYVSCYSPYAHMAKAFKGPFSFKDPKADP<br>MPKPAYAYHLNAVAFAKLLAEVGTERGIEHVLDDVDDVELDERGYVAALQL<br>RERGRHPVELVIDCTGFRGRIIREALDEPFEDYSDYLGNDRAMALQIPHTNP<br>EKLPPFTSSTALGAGWSWSVPLFNRVGTGYVFSSAHRTDDEARDEFLAHL<br>GAAAPPGSEPRVIPMRIGRSRRAWVKNCIAMGLSGGFIEPLESTAIHMIDQSI<br>RMLLFNMPSTDYEAPVRRRFNRLVDDLYGEVRDFICLHYRMGNREDSAYW<br>IDARTELKITDRLAENLELWQYQLPTQHDLESAQLFTHDTYQTVLFGKRCYE<br>NGYGAERLNTPREVRAGSWKAYLRDADIRFAKIVDYKGDHHRLLMALRGEL<br>EPGAARKTPAVVAARTGSCLKETPVLF
32	MRDMLRRFGLSERGVLAACDASFKHGIRFDDWSGPGSRCLHPFHRHALPR<br>DIAARWLASSGEPPFAAIASAQAALIAHERAPRPAGAPEFTGPVPYAYHLDA<br>EKFAEYLAGELTSRGVTRQSAHVTGVLREGETVTALRTEDGAPLSADLWVD<br>CTGFAGLLLPEEGRDWVSYADRLLCDRAVTMRVPEDDPAYTPAPFTRARA<br>LEAGWCWDIGLRHRRGRGYVYSSAHLSDDEAEAALRAEEGQHSAELPARV

	IHFTPGRRAAPWQGNVVALGLAAGFLEPLESTGLFLADFAARALVEMFPAA<br>PGLPMQPLARRYNALLAEMHDDLADFLALHYAVAQRRDTTFWRDAAKA<br>ALRSERLRELLELWQLRPPSFADFSYRYSPFSHTAWEFILLGMGWRPATAP<br>AGTTPCAAPAEALTRQMLDALPDHGTLLRHLAQACR
33	MNNNIKKIVIVGGGTAGWMAAASLAKYVNNSAEICLIESSDIATVGVGEATIP<br>NIVQFNRNLGIDETAFIKATQATFKLGIQFENWHPQNKQFFHPFADYGLKIDQ<br>VDFHHYLYRAQQTECSSQLADFSFACQLAKQYKFAQPHANPSTPLADYAY<br>AYHFDAGLYGQFLKKHSINAGVNHLIGTIEQVNLHPDTGFINNVALTDGQIVE<br>GEFFIDCSGFEGLLIEKALHTGYEDWSHWLFCDSALAVQTTRVNPPEPFTR<br>SIAQSAGWQWHIPLQHRSGNGYIYSSRFESDEQAKALLQTNINGEPLGEIRK<br>ITFKPGRRKQIWHKNCFALGLASGFLEPLESTSISLVQTAIAKLLTFFPDCSFN<br>QADINEVNRLHNLELENIRDFLILHYKLSARCDTPFWQACQNMAQQSHCHN<br>PHNYTRSRSRCSGVGRAPSPQSHSPSMLVRIQVLAAFEHIERRRVYALYLP<br>RI
34	MDIKKVAILGGGTAGWLTANHLGKALIGQGISITVIESPDIPTIGVGEGTVPMM<br>RETLKYFGISESQFIKECDATFKQSIKFVNWLDKTAHGEGNFYHHLFDFPFP<br>NNRDLTPFWLRTQHKSFAQTVSFQANVCEAKKAPKKISTPEYVGETTYAYH<br>LNAKKFAELLQNHAVNKFSVAHQVSTVIDATVNNTDIELIVTDTHGEQVFDFY<br>VDCSGFNCYLLGKKLNIPFVDMSHKLPIDKAITAQIPTEDDAEIPPYTIATAHE<br>AGWIWDIALKNRRGTGFVYSSNYLSDEEAKQKFERYLGQSLEGIVSRVIPM<br>KIGYREQFWQGNCVAIGLSQGFVEPLEATAILVTDFCARHLAERFPRSKSEL<br>PACQSRFNRNVKYAWDRVIDFIQLHYYLSDRQDSAFWLDWKNVDRLSDEL<br>KQKLAIWQNNPPLSTDFFSKFEVFDVENYLYVLYGMHYLTKPSYIDAKSFEQ<br>DQILAQQVE<br>DHSQKLIASLPSHRELIDKLARFDFSKL
35	MSHRVNQVVIIGGGTAGWLTAAVLSSQLQSTNKEAVQVTLVESPNIPILGVG<br>EGTWPNLRATLKQIGVSEAEFMKGCDATFKQGALFCNWMKSVNEQTHQY<br>YHPLNTVFHSSYEFSLAPYWLLGHSERSRYDHAVATQSLVCEAGLGPKEKT<br>TPEYQAVQEYSYHLNANKFARFLTKHCTEKLGVKYIQANVLKANRDGDDYV<br>TSVTTDSEQYDAICGDLFVDCSGTTPVLFEQTYHIPWVDISDVILNDTAIAMQ<br>VPYADPERDIASHTIMTAQDNGWIWDIGLYNRRGVGHVFSSKHTDIKTAEK<br>QLRDYIGQEAEGLTARVIPLKLGYREKFWHKNCVAIGMSAGFVEPLEASA<br>IYLYDAAAKMLADQFPRTRESMQYVEEKFNRSFNMRMIRTIDFIKLHYCISDR<br>DDSAYWRDNCSNKTIPKALQQRLLHWKEHPPTKYDFDNAWEPFNLDSYLY<br>VLYGMKYQTSLAHNQDGFIYSDKAKAKFRDVSMMADKLVTHLPKHRDLLKK<br>VYQFGFTKI
36	MTPTKISNVVIVGGGTSGWITAALLVKVLGRVINITLVESDEIGTIGVGEATIPP<br>IVQLNNALGISEKTFLAHTKGTFKLGIEFNDWGQLGDSYMHAFGGIGKSLPF<br>CDFHHLWLQSKQQGGNSDFWDFSLNYQAAMAGKFGPSQKIASTNIQGLEY<br>AYHFDAGLYAGLLSEFSQGLGVKRLEGKVTEVVVNQNNGDVSHLCLANGE<br>HLYGDLFIDCTGLKALLIEQTLNTGFEDYSHWLPCDRAVAVPCEKVEPIVPYT<br>RSTAHSCGWQWRIPLQHRTGNGLVYSSKYLSDEAAKAQLLNSLDGKPLGE<br>PKIIAFKTGRRRKQWHKNVVAIGLSNGFFEPLESTNIHLIQTAATRLIKFFPHF<br>GIKDAEVSEFNRQAQVEAEQMRDFIILHYKVNQKVDSPFWQACQRMEIPHT<br>LAQKIALFAQSGKVFREQDELFTEIAWQQVMLGQNLVPADIHPIATMLSPLQ<br>LSELMENLKTLVDHNVAGIVSHSDFLAQIAH
37	MNHKSRIVIVGGGTAGWLSAAKLAKELAANQPNGVQVTLLESSDIPTVGVG<br>EGTWPTIRKTLASIGIDEAEFIKETDASFKQATQFVNWKSDPISTNNTYYHLF<br>SSVSDSAQFNLAPYWLLGDKHTSYAQAVSAQDILCDKGLAPKLITNKPYEGI<br>QNYAYHLDAGKFATLLKRHCIEKLGVKHLIGNVVQVNTDEEGAIASLVTDTQ<br>GMLAADLYVDCTGFKSLLIGQALGVKFNSIADTLFVDNAIAIQMPYENDEADI<br>ASATLSTAQQAGWIWDIGLQSRRGTGHVYSSEFISHDEAEHTLRQYLGPKA<br>TDLPAKHIKMNCGYREKFWHKNCVAIGLSAAFVEPLEASAIFLIEASANMVA<br>ELFPINKALMQASAQTFNDSFNYRWRKTIDFIKLHYVLSNRSAPFWQANKRP<br>ESIPSSLSEMLNQWQHRPISKYDFAHVFEPFPQESYQYVLYGMGYHPDLA<br>MSQSRYANLDTAENLFGQVSQMRSNFEKHLTTNRALLNKVKKYGFQKL

38	MSDSKAPNVVVLGGGSAGWITACLLHRQWSEKGGSVTVVESPEIGIIGVGE GSTPQLKAFFDQLGIAESEWMPACNATYKLGIRFNGWSERAGFESYYHPFP GPIDLHSEPGFIGNCMLARHQIDVAAHPDDWFLATHLSEAGKSPIAAENFPF APSYGYHFDAYALGAFLRDWAVKRGVVHRPLKVTAVERADDGDIAALLCEG GERIEGDLFVDCSGFRAMLAEQELGATFLPFAENLFNDSAVVLPTGHGAPF KPQTEATAMRAGWRWSIPLTTRVGNGYVYSSKYISNEDAEAELRGALGLA GDGPEARFLKMKVGRLENSWTRNCLAVGLSQGFLEPLEATALHIVIVTAL EFAQAYEQGGYGPQHRDAFNASIAARYEGIRDYIVAHYRVNQRSDTQYWR DNAANDRLSNGLKTMMTAWFRHQDIHAANDEAYGGKRYYANASWHCLFA GYGTFPPQERMEAVPEGIRPADPAEARAMLEACAQNFPSYDPIAS
39	MNPTDLPPIVIAGGGTAGWMAAATLGRFAGRKVLLVESEAIGTVGVGEATIP QIRLFNAALGLDEAEFLRETQGTFKLAIEFAGWGGEGERYLHAFGTVGHGA GVLPFQHYWLRARQAGKAGPLSDYALNDVASRAGKMQMWQAEPGKPVP DMPWAYHFDAGLYAAYLRRYAEAHGVERVEGTIASVERDGESGDIAALVLD GERRIEGGVFVDCTGFRSLLLGGALGVEFMDWTQWLPCDRAVAVPCETKG EFTPYTRSTARKAGWQWRIPLQHRIGNGHVFCSDFMGEDEATKTLLANLD GAPTADPRVLKFTTGMRAQQWSHNCMALGLAGGFMEPLESTSIHLIQASIA RLLQMLPGAEVAPAMRAEFNRQAEFEWTRIRDFLILHYWANGREGEPFWD RCRAMDVPDTLSTKVETFRASGFVHREHEELFTEPGWLQVLIGQGVVPEN YNPIADAMPEVELTAMLARIAETNQRLVAAMPDHAPFLRAHIDPRKQAPTQQ RIPA
40	MHSMAEPVTSITVVGGGTAGWLAATYLRSKCAPKIAITLIESPNVPTVGVGE ATVPHMPVTLREMGIDDKEFFRRCNTSFKMGVMFRGWNVDHKGRPFDYM NPFAPPPKIDGVEAGHYFQAFGAGRRDYIQSFSPLMDLLDAGKCPLSFNAK NDPMPRAGYAYHLDAGAFAKMLAEVGVARGVEHVLDDVDGVELDDTGHV AALNLRALGRRPVQLVIDCTGFKGLVIREALGEPFENYSDYLANDRAMALPI PHADPEVLPAATSSTALGAGWSWSVPLYHRIGTGYVYSSAHRTDDAARDD FMAHLGDRAPKGAEPRVIPMRIGRMRRAWVKNCVALGLSGGFIEPLESTAI HMIDMGIRWLLQYFPSRDYEESTRAAYNRCADEMYNEVRDFICLHYRLGNR TDTDYWCDARGLKITDRLEGWLDLWQRQLPVEMDIGRANLFDHNTFQAVL LGKRVYENGWGAARLNRSFALSEKRWKAFLRDSAARNMAVVKAKADHRR ALRVLRGEATDGAVTRQSLGAVADEAVLF
41	MNNRLRKITIVGGGTAGWMTALILETELSRTSAPKDRPKICLIESPNIATVGV GEATVPRMPKTLRQAGISERAFFRETNASFKLGVKFCNWNKDAKGNRIDYV NPFAHGQLLDGLEPAEYFLRFGNGDRDYTQSIAPHDDLGRLCKGARPLGQ PEFEQRFGYAYHLDAVKFAGMLTKVCTKHGVEHIQDEVTSVELDEQGNVS HLMLEQRGRHDIEMVIDCTGFRGLIINQALGEPFMDYSDYLPNDRAMALQID HPDPEKIESLTRSTALGAGWTWRVPLYNRVGTGYVFSSAHRTDDQAADEY LEWLGDSGKGATPRIIPMRIGRVRNAWVKNCVAIGLAGGFIEPLESTAIHMID HAVRWLTEHMPTRDIAPSLRTRYNRQMDKLYDEVLEFICLHYRLGNRTDDQ YWIDARTEMKIPDRLAENLELWQHRLPMSHDIEFATLFDYRVYQTVLLGKQV YDTGYGPGIRDRLRPLKKPIWFQWWKGAKADLAQILKAMPDHKTLLRDIRG ELDQPGFGMAAAMKPTVPMPGAAPVPWAIQNMPSFSEIESGQKDLQLF
42	MNNTIKKVVIAGGGTAGWMAAASLSKLIGKNLEITLIESDEIPTVGVGEATIPP LILLHQLLDMDEKEFMKAVHGTFKLGICFENWRDVNEDYIHSFGYTGKDCW AANFLHFWLKGLKEGISKDYGVYCTELIAAQQNKFAVLQSGALNYAYHIDAG RYAALLRKIAEKHGTTRQEGRIDSVATNEHTGYIESVTLASGQRIEGDLFIDC TGFRGLLIEQTLHTGYDDWSHWLPCDSAVAVQTRSVGPPIPYTRSIARDAG WQWRIPLQSRVGNGLVFCSRYLSDDEAIQTILNNVEGETITDPRVIKFRTGQ RRKHWNKNCVAMGLASGFIEPLESTSIHLIQKAVTRLIQNFPFEGIHESEI KEFNTQMDADTENIRDFIILHYHVTNREDTRFWRHCKNMSIPDSLQHRIDLF KETGRVFKSPNDLFVENSWIQVMLGQGLMPKQYHTIVDEMSDEELRKFMQ GAELSVQRTVSQLPSHQAFIDHYCKADLGTAGEHAMPLNPQSGVRAAS
